# Novel tools to quantify total, phospho-Ser129 and aggregated alpha-synuclein in the mouse brain

**DOI:** 10.1038/s41531-024-00830-y

**Published:** 2024-11-08

**Authors:** Benjamin Guy Trist, Courtney Jade Wright, Alejandra Rangel, Louise Cottle, Asheeta Prasad, Nanna Møller Jensen, Hjalte Gram, Nicolas Dzamko, Poul Henning Jensen, Deniz Kirik

**Affiliations:** 1 Charles Perkins Centre, School of Medical Sciences, Faculty of Medicine and Health, The University of Sydney, Camperdown, Australia; 2 Brain and Mind Centre, School of Medical Sciences, Faculty of Medicine and Health, The University of Sydney, Camperdown, Australia; 3 Brain Repair and Imaging in Neural Systems (BRAINS), Department of Experimental Medical Science, Faculty of Medicine, Lund University, Lund, Sweden; 4 Department of Biomedicine, Aarhus University, Aarhus, Denmark; 5 Danish Research Institute of Translational Neuroscience - DANDRITE, Aarhus University, Aarhus, Denmark; 6 Present Address: Melanoma Institute Australia, Sydney, NSW Australia

**Keywords:** Parkinson's disease, Neurodegeneration

## Abstract

Assays for quantifying aggregated and phosphorylated (S129) human α-synuclein protein are widely used to evaluate pathological burden in patients suffering from synucleinopathy disorders. Many of these assays, however, do not cross-react with mouse α-synuclein or exhibit poor sensitivity for this target, which is problematic considering the preponderance of mouse models at the forefront of pre-clinical α-synuclein research. In this project, we addressed this unmet need by reformulating two existing AlphaLISA^®^ SureFire^®^ Ultra™ total and pS129 α-synuclein assay kits to yield robust and ultrasensitive (LLoQ ≤ 0.5 pg/mL) quantification of mouse and human wild-type and pS129 α-synuclein protein. We then employed these assays, together with the BioLegend α-synuclein aggregate ELISA, to assess α-synuclein S129 phosphorylation and aggregation in different mouse brain tissue preparations. Overall, we highlight the compatibility of these new immunoassays with rodent models and demonstrate their potential to advance knowledge surrounding α-synuclein phosphorylation and aggregation in synucleinopathies.

## Introduction

Synucleinopathies are a group of clinically heterogeneous neurodegenerative disorders characterized by a unifying pathological feature: the abnormal deposition of Lewy pathology within neuronal and/or glial cell bodies and processes^1^. Within this broader classification, the main subtypes are Parkinson’s disease, representing the commonly seen clinical presentation, and two rare variants: dementia with Lewy bodies (DLB) and multiple system atrophy (MSA), the latter manifesting as two distinct subgroups exhibiting predominant symptoms of either cerebellar ataxia or parkinsonism. There are currently no disease-modifying treatments capable of slowing or halting cell death in any of these disorders, however, the abundance of Lewy pathology in brain regions undergoing significant functional decline and degeneration has made it a focal point for the development of such interventions.

The structural morphology and composition of Lewy pathology are highly heterogeneous across synucleinopathies^2^, as well as within and between different brain regions of each disorder^3,4^, complicating attempts to understand mechanisms driving its formation. Lewy pathology broadly comprises spherical Lewy bodies (LBs) and pale bodies, as well as filamentous Lewy neurites (LNs), which are composed of dysmorphic organellar components, lipid membranes^5^ and many hundreds of proteins^6^. Despite such incredible variability in composition, α-synuclein protein is invariably enriched in all forms of Lewy pathology and is suggested to play a key role in its propagation^3^. The leading hypothesis proposes that intraneuronal α-synuclein misfolds, oligomerizes and transforms into β-sheet-rich amyloid fibrils^7^, which form the foundation of neuronal Lewy pathology. Furthermore, intermediate misfolded forms of α-synuclein are thought to be transferred to neighboring neuronal and glial cells upon their release^8,9^. The molecular pathways governing α-synuclein misfolding, aggregation, fibrillization, transmission and Lewy pathology formation, however, remain poorly understood.

Post-translational modifications (PTMs) are chemical modifications to amino acid residue side chains of a protein that impact biophysical properties such as surface charge, hydropathy, size and conformation, which can in turn influence protein misfolding, aggregation, subcellular localization, activity and degradation. A large number of PTMs are reported for α-synuclein protein—phosphorylation, ubiquitination, nitration, acetylation, truncation, SUMOylation, glutathionylation and glycosylation^10^, with phosphorylation of serine residue 129 (pS129) attracting particular interest due to its high abundance within Lewy pathology^11^. While this has led to the development of several robust assays capable of quantifying human wild-type (WT) and pS129 α-synuclein in brain tissue, blood and cerebrospinal fluid^11–16^, assay cross-reactivity for mouse WT and pS129 α-synuclein is poor^12,16^. This is problematic given the preponderance and implementation of mouse models in research examining the physiological and pathological roles of α-synuclein in Lewy pathology, and the potential utilization of these models in therapeutic development.

To address this critical unmet need, we worked with the manufacturers of the AlphaLISA^®^ SureFire^®^ Ultra™ Total (PerkinElmer^®^, #ALSU-TASYN) and Phospho-α-Synuclein (Ser129; PerkinElmer^®^, #ALSU-PASYN) Detection Kits to adapt their formulations and ensure compatibility with both human and mouse α-synuclein. Comprehensive characterization of these reformulated assays demonstrates that they are capable of robust and highly sensitive (~0.1–0.5 pg/mL) quantification of both human and mouse WT and pS129 α-synuclein in vitro, in complex cell lysates, as well as brain tissue extracts. Furthermore, we complemented these assays with the aggregated α-synuclein ELISA assay from BioLegend^®^ to provide the first demonstration of alterations to the total abundance, phosphorylation, aggregation, and subcellular compartmentalization of mouse α-synuclein within the brains of WT mice following inoculation with WT mouse α-synuclein pre-formed fibrils (PFFs).

## Results

### Selection and validation of new antibody pairs for reformulated SureFire Ultra pS129 and total α-synuclein assays using purified protein standards

**Fig. 1:**
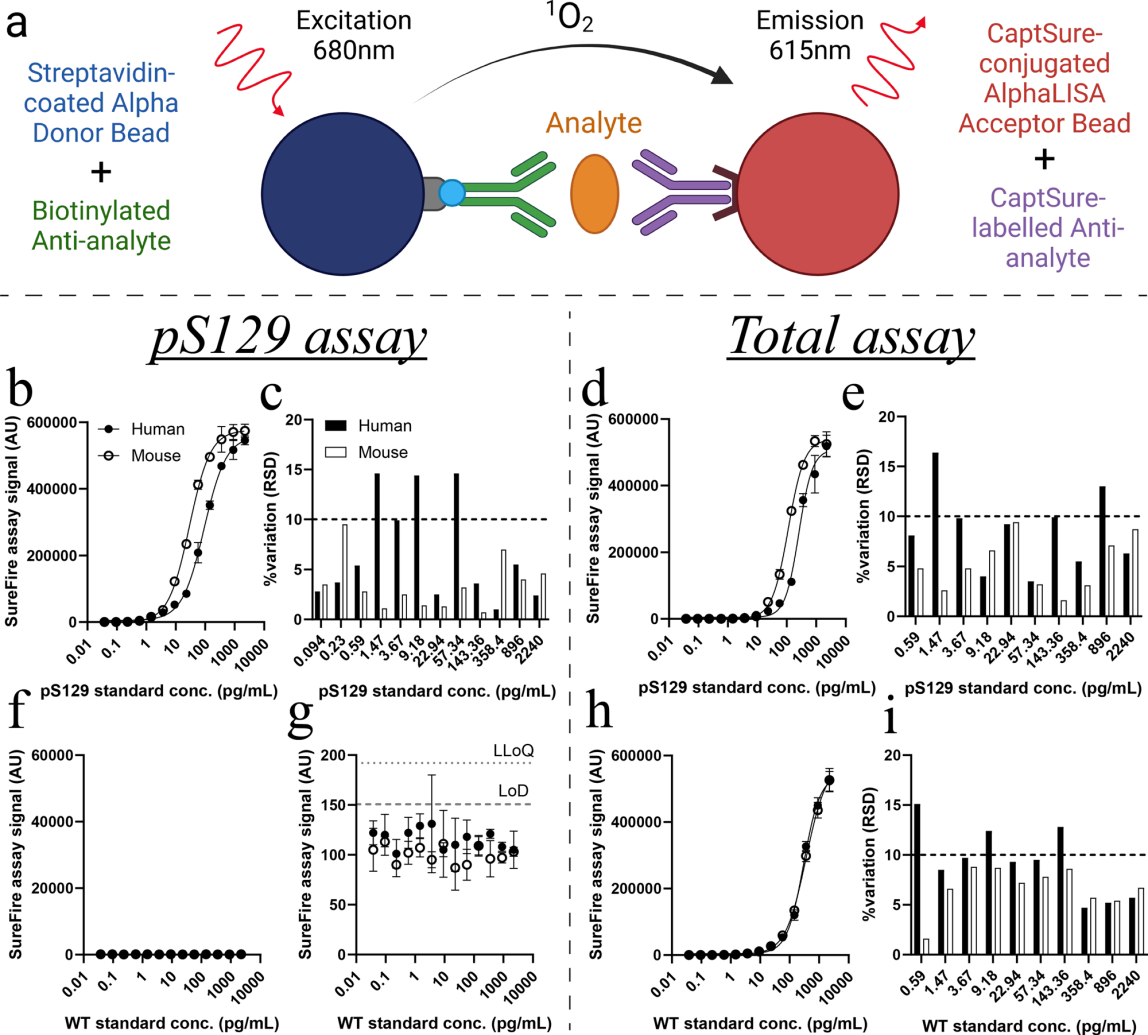
Characterization of new pS129 and total α-synuclein SureFire Ultra assays using purified protein standards. AlphaLISA SureFire Ultra assays are designed using two antibodies against a target protein, which are differentially tagged to ensure their selective conjugation to one of two types of Alpha beads; donor beads or acceptor beads. The binding of both antibodies to an analyte brings donor and acceptor beads into very close proximity, which enables donor beads to activate acceptor beads following sample photoexcitation to produce the assay signal (**a**). Five new formulations of the pS129 (left) and total (right) α-synuclein assay were characterized using purified human (Hu) and mouse (Ms) pS129 α-synuclein standards (**b**–**e**), as well as human and mouse wild-type α-synuclein standards (WT; **f**–**i**). Data presented above represent only the most sensitive formulation of each assay. Associated data for all tested formulations are contained in Supplementary Tables 2, 3. Inter-assay variation (RSD) was calculated from three individual standard curves constructed and measured on separate days (**c**, **e**, **i**). The limit of detection (LoD) and lower limit of quantification (LLoQ) were defined as 3 and 6 standard deviations above the mean of the blank, respectively. Data in (**b**, **d**, **f**, **h**) represent mean ± standard deviation, while % variation data in (**c**, **e**, **i**) are only presented for standard concentrations detected above the LLoQ. For all standard curves; number of independent experiments (*N*) = 3, number of replicates in each experiment (*n* = 3). AU arbitrary units.

Prior to this project, we discovered that the existing SureFire Ultra pS129 α-synuclein assay (#ALSU-PASYN-A, PerkinElmer) did not recognize human pS129 α-synuclein, while the existing total α-synuclein assay (#ALSU-TASYN-A, PerkinElmer) was unable to detect mouse pS129 or mouse WT α-synuclein (Supplementary Table 1). We therefore aimed to improve cross-species detection in both of these assays by screening five new formulations of each assay against purified human and mouse pS129 and WT α-synuclein recombinant protein standards (Fig. 1a). Each new formulation contained different immunocapture antibody pairings, which were chosen based on their cross-reactivity for mouse and human α-synuclein isoforms in previous immunocapture or immunostaining experiments conducted by our group and others^11,12,17^. Accordingly, both species of pS129 α-synuclein were robustly detected above background signal produced from assay lysis buffer by three new pS129 α-synuclein assay formulations (Fig. 1b, c) and one new total α-synuclein assay formulation (Fig. 1d, e). Neither species of WT α-synuclein were detected by any pS129 assay formulation (Fig. 1f, g), yet were detected by the same total assay formulation as that recognizing both phosphorylated proteins (Fig. 1h, i). Complete assay characterization data for all assay formulations using purified protein standards are provided in Supplementary Tables 1–3.

The most sensitive formulation of each assay yielded 5–20-fold improvements in sensitivity compared with previous SureFire Ultra α-synuclein assay formulations (Supplementary Table 1). The most sensitive new pS129 assay exhibited a limit of detection (LoD) between 0.02–0.04 pg/mL and a lower limit of quantification (LLoQ) between 0.04 and 0.08 pg/mL for both human and mouse pS129 α-synuclein species. Its linear dynamic range was between 0.1 and 143.4 pg/mL for all standards (Supplementary Fig. 2) and the average inter-assay variability was 5% (range 0.7–14.6%). Similarly, the new total assay formulation exhibited a LoD between 0.04 and 0.09 pg/mL and a LLoQ between 0.1 and 0.5 pg/mL for all standards. Its linear dynamic range was between 0.2 and 896 pg/mL for all standards except mouse pS129 α-synuclein (0.2–358 pg/mL; Supplementary Fig. 2), with an average inter-assay variability of 8.0% (range 1.6–15.0%).

### Assessment of reformulated assay sensitivity and selectivity in complex biological matrices

**Fig. 2:**
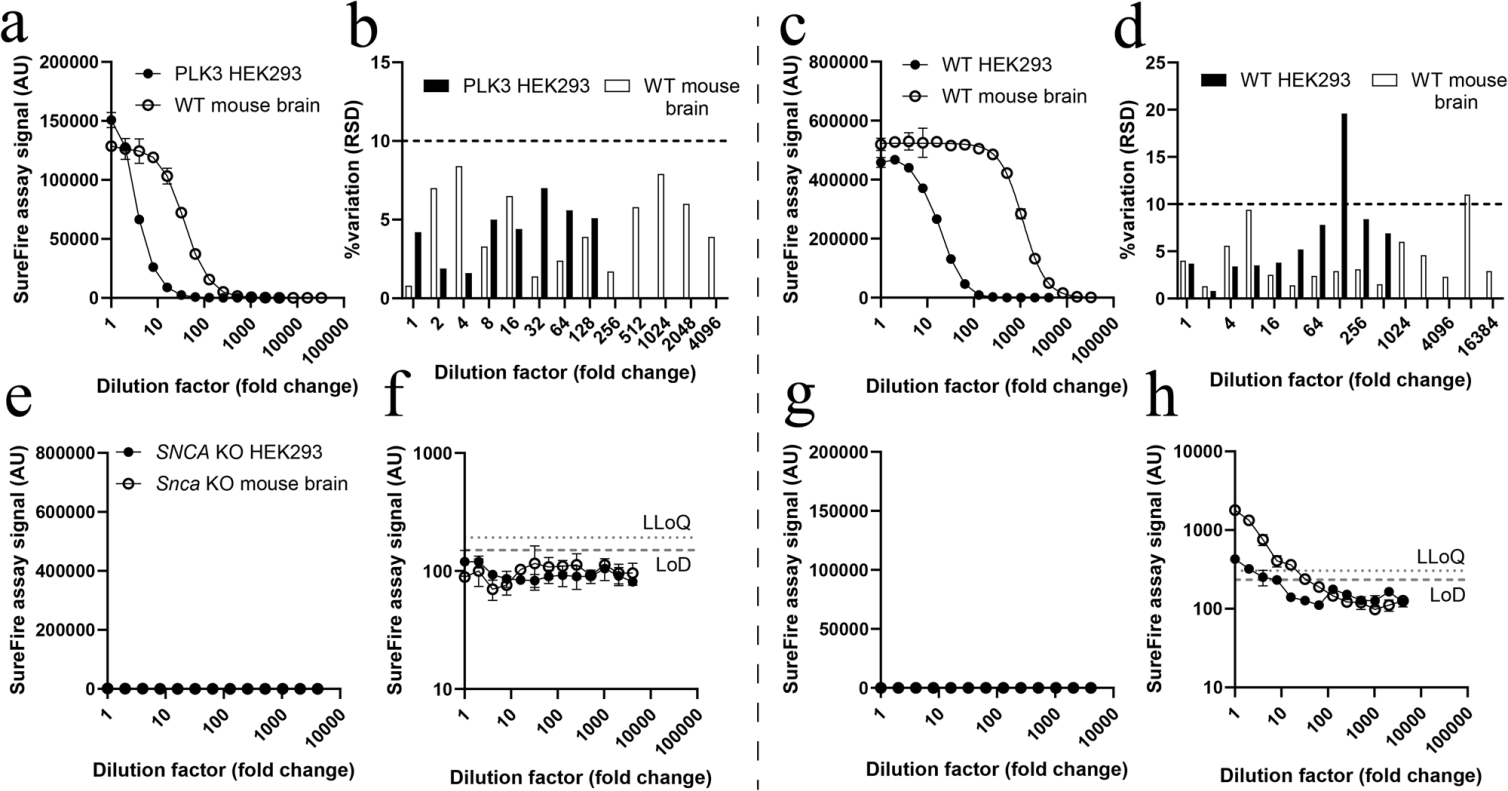
Characterization of new pS129 and total α-synuclein SureFire Ultra assays using mouse brain tissue extracts and HEK293 cell lysates. The most sensitive new pS129 α-synuclein assay (left) was characterized using wild-type (WT) mouse brain tissue extracts and PLK3-transfected HEK293 cell lysates (**a**, **b**), while testing of the most sensitive total α-synuclein assay (right) utilized WT mouse brain tissue extracts and WT HEK293 cell lysates (**c**, **d**). Extracts and lysates were serially diluted using assay buffer. Associated assay data for all formulations tested are contained in Supplementary Table 4. Inter-assay variation (RSD) data presented in (**b**) and (**d**) were calculated from three individual dilution curves constructed and measured on separate days, with data only shown for extract/lysate dilutions above the lower limit of quantification (LLoQ). Phospho-S129 (**e**, **f**) and total (**g**, **h**) assay specificity were also assessed using *SNCA* knock-out (KO) HEK293 cell lysates and *Snca* KO mouse brain tissue extracts. Protein concentrations in each sample prior to serial dilution were as follows: WT mouse brain supernatant, 4.93 mg/mL; KO mouse brain supernatant, 5.48 mg/mL; WT HEK293 supernatant, 4.77 mg/mL; KO HEK293 supernatant, 5.6 mg/mL; PLK3-HEK293 supernatant, 6.55 mg/mL. The limit of detection (LoD) and LLoQ were defined as described in Fig. 1. Data in (**a**, **b**, **g**, **h**) represent mean ± standard deviation. For all standard curves; number of independent experiments (*N*) = 3, number of replicates in each experiment (*n* = 3). AU arbitrary units.

Screening new assay formulations in vitro informed on the compatibility of new antibody pairs and their selectivity for mouse and human α-synuclein isoforms in isolation, however, these parameters can differ in complex biological matrices if target antigen conformations vary due to interactions with other cellular components. We cross-validated the in vitro sensitivity and selectivity of new assay formulations for both species of WT and pS129 α-synuclein in complex biological matrices by applying the most sensitive formulation of each assay to WT mouse brain tissue and HEK293 cell extracts. These assays will subsequently be referred to as the reformulated total and pS129 α-synuclein assays, which are now commercially available through Revvity (total, #ALSU-TASYN-B; pS129, #ALSU-PASYN-B). While pS129 α-synuclein is abundant in the WT mouse brain, it exists in comparatively low abundance in WT HEK293 cells^12^, hindering assessment of pS129 α-synuclein assay performance under baseline conditions in this cell line. To address this challenge, we transfected WT HEK293 cells with a vector construct expressing polo-like kinase 3 (PLK3) under a human CMV promoter^18^, dramatically increasing phosphorylation of α-synuclein at the S129 residue. Mirroring in vitro data, the reformulated pS129 α-synuclein assay subsequently robustly detected mouse and human pS129 α-synuclein in WT mouse brain and PLK3-transfected HEK293 cell extracts (Fig. 2a, b), while the reformulated total α-synuclein assay detected mouse and human α-synuclein in WT mouse brain and HEK293 cell extracts (Fig. 2c, d). Both assays again exhibited low inter-assay variability in these matrices (average 5.0%, range 0.8–19.6%).

To assess non-specific binding of assay reagents to other matrix components, we applied our reformulated pS129 and total α-synuclein assays to *Snca* knock-out (KO) mouse brain tissue extracts and *SNCA* KO HEK293 cell lysates diluted serially with assay lysis buffer. We did not detect any signal above the LLoQ for any dilution of either KO matrix using the pS129 assay, suggesting high specificity of this assay for pS129 α-synuclein (Fig. 2e, f). Application of the total assay to both KO matrices produced a very small signal (<0.3–0.4% of max assay signal for corresponding WT matrices) above the LLoQ in the most concentrated sample dilutions tested (Fig. 2g, h), suggesting that one or both antibodies within this formulation possess a very low affinity for one or more components of both matrices aside from α-synuclein. We determined that the minimum required dilution (MRD) to successfully ameliorate these background signals was ~24-fold in *Snca* KO mouse brain tissue extracts and <8-fold in *SNCA* KO HEK293 cell lysates. Importantly, both of these MRDs are lower in magnitude than the dilution factors required to reach the linear dynamic range in corresponding WT mouse brain (256-fold) and HEK293 cell (>8-fold) extracts, making it highly unlikely that these minor matrix effects distort true positive assay signals. Complete assay characterization data for mouse brain extracts and HEK293 cell lysates are provided in Supplementary Table 4.

### Evaluation of matrix effects and their impact on reformulated assay performance

A key step in validating any ligand-binding assay is evaluating whether the immunoaffinity characteristics of the assay differ when performed in calibrator matrix (assay lysis buffer) compared with sample matrices (mouse brain tissue or HEK293 cell extracts). Differences in these characteristics between matrices, known as “matrix effects”, commonly arise from specific or non-specific interactions between matrix components and capture/detection reagents or the target antigen itself, which can diminish assay accuracy and sensitivity. We conducted parallelism and spike-in experiments to confirm the suitability of our calibrator matrix (assay lysis buffer) for measuring endogenous α-synuclein in mouse brain tissue and human cell extracts.

**Fig. 3:**
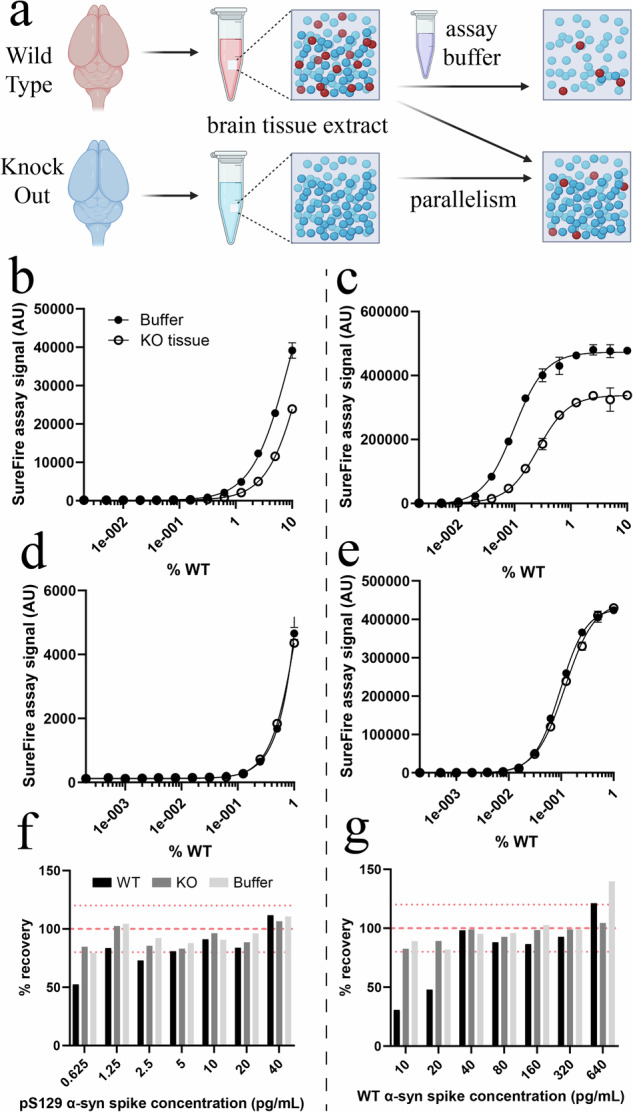
Evaluation of matrix effects and their impact on SureFire Ultra assay performance. The magnitude of matrix effects produced by mouse brain tissue was first evaluated in new assay formulations using parallelism experiments (**a**). Matrix effects were measured for the pS129 (left) and total (right) assay using wild-type (WT) and *Snca* knock-out (KO) mouse brain tissue extracts that had both been diluted 10-fold (**b**, **c**) and 100-fold (**d**, **e**). Phospho-S129 (**f**) and WT (**g**) mouse α-synuclein were also spiked into WT and *Snca* KO mouse brain tissue extracts diluted 2000-fold and 100-fold, respectively, as well as assay buffer (AB), and spike recovery assessed using standard curves generated for purified mouse WT and pS129 α-synuclein. Measured spike concentration was calculated using the increase in assay signal above baseline signal generated by assay buffer, WT extract or KO extract, which was then expressed as a % of the intended spike concentration (% recovery). Wild-type α-synuclein was spiked into samples at 10–640 pg/mL and measured using the new total α-synuclein assay, while pS129 α-synuclein was spiked in at 0.625–40 pg/mL and measured using the new pS129 α-synuclein assay. Dotted red lines in (**f**) and (**g**) represent 80% and 120% spike recovery, while dashed red lines in these panels represent 100% spike recovery. Data in (**b**–**e**) represent mean ± standard deviation. For each panel; number of independent experiments (*N*) = 1, number of replicates in each experiment (*n* = 3). Associated data are contained in Supplementary Table 5–7. AU arbitrary units.

In parallelism experiments, WT and α-synuclein KO mouse brain tissue extracts were first diluted 10-fold and 100-fold, constituting dilution factors above and below the MRD (24-fold) previously determined to ameliorate non-specific assay signals in *Snca* KO mouse brain extracts (Fig. 2h). Pre-diluted WT matrices were then serially diluted using either assay lysis buffer or the corresponding dilution of α-synuclein KO matrix (Fig. 3a). While serial dilutions in assay lysis buffer progressively reduced the concentration of all components of the study matrix, dilutions made using corresponding KO matrices ensured all components remain constant throughout the sample-dilution response curve except α-synuclein, preserving any matrix effects throughout the dilution series. If matrix effects are indeed absent beyond the MRD, the sample-dilution response curves for WT matrix diluted in assay lysis buffer and KO matrix should coincide. We observed significant sample-dilution response curve shifts in 10-fold-diluted WT mouse brain extracts diluted in KO matrix compared with assay buffer for both reformulated assays (Fig. 3b, c), which disappeared when extracts were diluted 100-fold (Fig. 3d, e). These distortions were markedly different from false-positive signals observed in neat α-synuclein KO matrices using the total α-synuclein assay (Fig. 2h), with sample-dilution response curve shifts demonstrating a dampening of assay signal in 10-fold-diluted matrices compared with 100-fold-diluted matrices. Parallelism experiments were also performed in WT and *SNCA* KO HEK293 cell extracts, revealing a lower magnitude matrix effect that was ameliorated by diluting extracts ≥20-fold (Supplementary Fig. 3).

For spike-in experiments, WT and α-synuclein KO mouse brain extracts were inoculated with known quantities of mouse WT and pS129 α-synuclein, before spike concentrations measured to assess the accuracy of α-synuclein quantification in these samples. Knock-out mouse brain extracts were diluted 100-fold to prevent matrix effects, while WT mouse brain extracts were further diluted to 2000-fold to ensure their α-synuclein concentration was on the lower end of the assays’ linear dynamic range. WT α-synuclein spikes were measured using the total assay and pS129 α-synuclein spikes measured using the pS129 assay. We observed proportionate increases in assay signal with increasing spike concentrations for pS129 and total assays, which did not differ significantly between matrices. Spike recovery from assay lysis buffer and mouse brain matrices averaged 80–92% for the pS129 assay (Fig. 3f) and 79–94% for the total assay (Fig. 3g), with notably lower spike recovery observed at lower spike concentrations. Spike recovery was especially poor for low spike concentrations made in WT mouse brain matrix, which we attribute to higher assay background signals produced by endogenous α-synuclein in this matrix. Complete assay characterization data for parallelism and spike-in experiments is provided in Supplementary Tables 5–7.

### Intrastriatal PFF inoculation differentially alters α-synuclein abundance and phosphorylation across the WT mouse brain

**Fig. 4:**
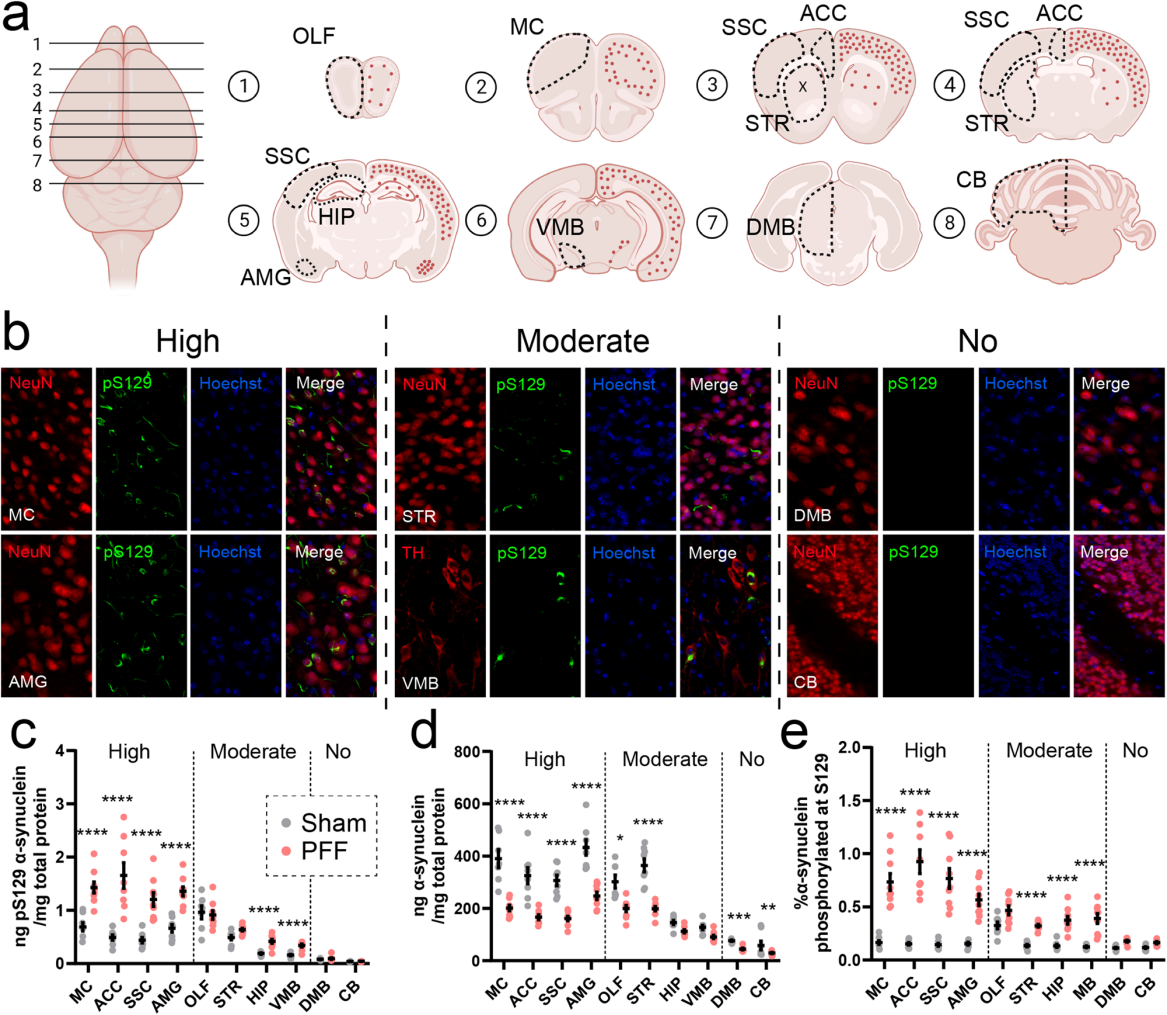
Profiling changes to the regional abundance of total and pS129 α-synuclein in the PFF mouse brain. The deposition of pS129 α-synuclein was highly heterogeneous throughout the brains of mice inoculated with mouse α-synuclein PFFs (*n* = 3; **a**, **b**). **a** The representative burden of pS129 α-synuclein deposits (red dots) within the PFF mouse brain, which was assessed using pS129 α-synuclein immunofluorescent staining (EP1536Y, Abcam) of fixed 30 µm free-floating coronal brain tissue sections from 3-month-old PFF mice (**b**). Full immunofluorescent characterization of all investigated brain regions in sham (*n* = 3) and PFF (*n* = 3) mice is presented in Supplementary Fig. 4. Phosphorylated S129 (**c**) and total (**d**) α-synuclein were quantified in fresh tissue extracts from sham (*n* = 8) and PFF (*n* = 9) mouse brain regions exhibiting high (MC, ACC, SSC, AMG), moderate (OLF, STR, HIP, VMB) or no (MRN, CB) pS129 α-synuclein pathology using the reformulated SureFire Ultra pS129 and total α-synuclein assays. The proportion of pS129 α-synuclein (**e**) was calculated by normalizing pS129 α-synuclein levels to the total amount of α-synuclein in these same tissue extracts. Datapoints in (**c**–**e**) represent the mean analyte quantity for a given sample, which was calculated from triplicate measurements of that sample in the same assay plate. Error bars in (**c**–**e**) represent mean ± standard error of the mean for that sample group. Data in (**c**–**e**) were generated from one independent experiment (*N*) performed in triplicate. *p < 0.05, **p < 0.01, ***p < 0.001, ****p < 0.0001; two-way ANOVA with Sidak’s multiple comparisons post-hoc tests, complete details of statistical tests are presented in Supplementary Table 8. ACC anterior cingulate cortex, AMG amygdala, AU arbitrary units, CB cerebellum, DMB dorso-medial midbrain, HIP hippocampus, MC motor cortex, OLF olfactory bulb, SSC somatosensory cortex, STR striatum, VMB ventral midbrain.

The α-synuclein PFF model has become a widely used animal model of Parkinson’s disease, involving the central or peripheral injection of recombinant α-synuclein protein fibrils^19^ to trigger widespread α-synuclein phosphorylation and aggregation throughout the brain. However, tools to accurately quantify the concentration of these abnormal species of α-synuclein in the rodent brain are lacking. To determine whether our reformulated α-synuclein assays address this gap, we first performed bilateral striatal injections of mouse α-synuclein PFFs (5 µg/hemisphere) in WT mice to induce significant synucleinopathy, with corresponding PBS-injected mice (sham) serving as controls. Immunohistochemical profiling of pS129 α-synuclein was then conducted in fixed brain tissues from PFF (Fig. 4a, b) and sham (Supplementary Fig. 4) mice 3 months post-injection, and qualitative observations made to identify regions exhibiting high, moderate or no pS129 α-synuclein pathology. Cortical and limbic regions such as the motor cortex (MC), anterior cingulate cortex (ACC), somatosensory cortex (SSC) and amygdala (AMG) exhibited a high pathological burden in mouse α-synuclein PFF-treated mice, with other regions of these same brains such as the olfactory bulb (OLF), striatum (STR), hippocampus (HIP) and ventral midbrain (VMB) only possessing moderate pS129 α-synuclein pathology. All sham-treated mouse brain regions, as well as select regions of PFF-treated mice (cerebellum, CB; dorso-medial midbrain, DMB), showed minimal or no pathology. Following histological profiling, we applied both reformulated SureFire Ultra α-synuclein assays to whole tissue extracts from these same regions of the sham and PFF mouse brain to provide the first quantitative measurements of pS129 and total α-synuclein across the mouse brain following PFF inoculation.

We observed substantial heterogeneity in absolute levels of pS129 and total α-synuclein across the sham mouse brain, with the highest levels of both species present in cortical and limbic regions that are susceptible to developing substantial pS129 α-synuclein pathology upon intrastriatal PFF inoculation (0.44–0.69 ng pS129 α-synuclein/mg total protein, 306.7–433.8 ng total α-synuclein/mg total protein). By contrast, phosphorylated and total α-synuclein levels were much lower in sham mouse regions that are spared from pS129 α-synuclein pathology following PFF inoculation, such as the DMB and CB (0.04–0.08 ng pS129 α-synuclein/mg total protein, 58.1–76.7 ng total α-synuclein/mg total protein) (Fig. 4c, d). Despite this heterogeneity, the proportion of α-synuclein S129 phosphorylation was remarkably consistent (0.12–0.16%) across all investigated brain regions in sham mice (Fig. 4e). The olfactory bulb constituted an exception to this observation, possessing a 2.4-fold higher proportion of α-synuclein S129 phosphorylation compared with all other sham mouse brain regions (0.33%; *p* < 0.0001 vs all other brain regions, One-way ANOVA with Dunnett’s multiple comparisons post-hoc tests, *q* = 7.4–9.8, DF = 70), consistent with previous data^20,21^.

In whole tissue extracts from PFF-inoculated mice, absolute levels of pS129 α-synuclein were significantly higher in regions of high pathological burden (1.2–1.6 ng/mg total protein) compared with sham mice (Fig. 4c), as was the proportion of α-synuclein S129 phosphorylation (0.57–0.93%) (Fig. 4e). This was accompanied by a 43–49% reduction in total α-synuclein levels in these regions of PFF mice compared with sham mice (Fig. 4d). Similar trends were identified in PFF mouse brain regions exhibiting moderate pathological burden, albeit to a lesser magnitude (0.3–0.9 ng pS129 α-synuclein/mg total protein; 0.32–0.47% pS129 α-synuclein; 23–45% reduction in total α-synuclein) (Fig. 4c–e).

α-Synuclein is predominantly expressed within pre-synaptic nerve terminals^22,23^, hence the reduction in total α-synuclein in regions of high-moderate pS129 α-synuclein pathology may reflect reduced synaptic terminal densities in these regions. To address this possibility, we quantified levels of the pre-synaptic vesicular protein synaptophysin, which constitutes a robust proxy for pre-synaptic architecture and abundance that has been used previously to inform on synaptic density in α-synuclein PFF models^24,25^. Synaptophysin levels were unchanged in the OLF, ACC, AMG, STR and CB between PFF and sham mice (Supplementary Fig. 5), suggesting reductions in total α-synuclein do not derive from reduced synaptic density in PFF mice.

### Altered subcellular distribution of total and pS129 α-synuclein in PFF mice

**Fig. 5:**
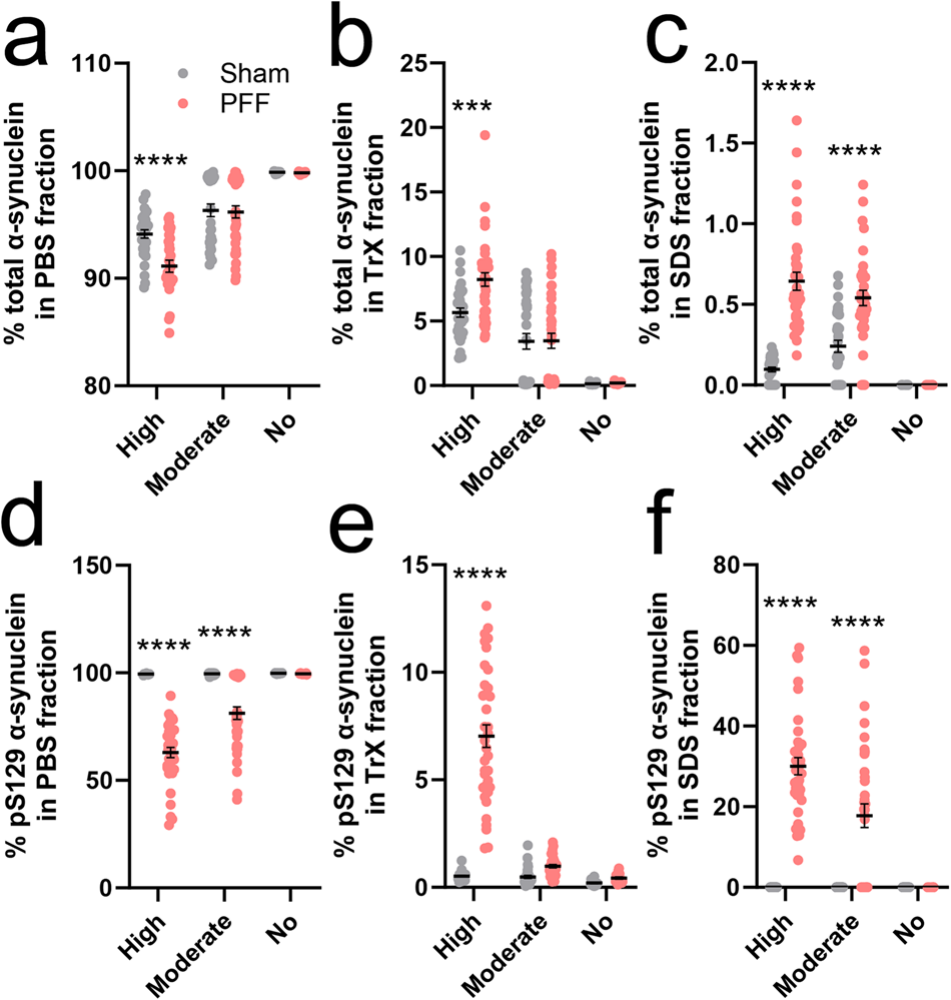
Characterizing alterations to the compartmental distribution of total and pS129 α-synuclein in the PFF mouse brain. Fresh tissues from sham (*n* = 8) and PFF (*n* = 9) mouse brain regions exhibiting high (MC, ACC, SSC, AMG), moderate (OLF, STR, HIP, VMB) or no (MRN, CB) pS129 α-synuclein pathology following PFF inoculation were fractionated sequentially using saline solution containing protease and phosphatase inhibitors (PBS), followed by the same buffer containing 0.5% Tween-20 and 0.5% Triton X-100 (TrX), and finally with a TrX solution that had 2% SDS added to it (SDS). Total (**a**–**c**) and pS129 (**d**–**f**) α-synuclein were then quantified in these fractions using the new SureFire Ultra assays. Datapoints represent the mean analyte quantity for a given sample, which was calculated from triplicate measurements of that sample in the same assay plate. Error bars represent mean ± standard error of the mean for that sample group. Data in all panels were generated from one independent experiment (*N*) performed in triplicate. ***p < 0.001, ****p < 0.0001; two-way ANOVA with Sidak’s multiple comparisons post-hoc tests, complete details of statistical tests are presented in Supplementary Table 8. Associated data are contained in Supplementary Tables 9, 10, with absolute amounts of total and pS129 α-synuclein contained within Supplementary Tables 11, 12. AU arbitrary units.

Changes to the intracellular distribution of native and pS129 α-synuclein have been previously reported in the PFF mouse brain^26,27^, however, no study has been able to measure these changes due to the absence of quantitative α-synuclein assays. We addressed this knowledge gap by quantifying the distribution of total and pS129 α-synuclein in PBS-soluble, Triton X-100-(TrX-)soluble, and SDS-soluble brain tissue fractions^28^ from PFF and sham mouse brain regions using our reformulated α-synuclein assays (Supplementary Tables 9–12). The proportion of total α-synuclein contained within each of these fractions was highly heterogeneous between sham mouse brain regions (Fig. 5a–c). While the majority (94.2–99.9%) was localized to the PBS fraction (Fig. 5a), TrX-soluble α-synuclein was enriched 42-fold (5.7%) in cortical regions prone to developing severe synucleinopathy, and 26-fold (3.4% total) in healthy brain regions that were susceptible to developing moderate synucleinopathy upon PFF inoculation (OLF, STR, HIP, VMB; Fig. 5b). Extremely little α-synuclein (<0.24%) was found to exist naturally in the SDS fraction in sham mice (Fig. 5c).

Despite significant alterations to total α-synuclein abundance in some brain regions following PFF inoculation, the proportion of total α-synuclein in each of the three tissue fractions was only marginally different in regions of severe pathological burden in PFF mice compared with sham mice (MC, ACC, SSC, AMG; Fig. 5a–c). In these regions, intrastriatal PFF inoculation promoted a redistribution of 3.1% of PBS-soluble α-synuclein (91.14% total) to TrX (2.55% increase, 8.22% total) and SDS fractions (0.55% increase, 0.64% total). Total α-synuclein distribution between the three fractions in remaining brain regions (OLF, STR, HIP, VMB, MRN, CB) was near identical between PFF and sham mice (Fig. 5a–c), with 93.2-99.4% localized to the PBS fraction, 0.2–6.8% found in the TrX fraction and <0.5% contained within the SDS fraction.

Contrast to total α-synuclein, the proportion of pS129 α-synuclein in each of the three tissue fractions was remarkably similar between sham mouse brain regions (Fig. 5d–f), being almost exclusively (99.5–99.8%) found in the PBS fraction (Fig. 5d). This became significantly disrupted upon PFF inoculation, with 17.8% of pS129 α-synuclein subsequently contained in the SDS fraction in brain regions exhibiting moderate synucleinopathy, which rose to 30.1% in regions exhibiting severe pS129 α-synuclein pathology (Fig. 5f). Comparatively little (1.0–7.0%) pS129 α-synuclein was found in the TrX fraction in these brain regions (Fig. 5e).

### Aggregation of membrane-bound α-synuclein occurs largely in the absence of S129 phosphorylation

**Fig. 6:**
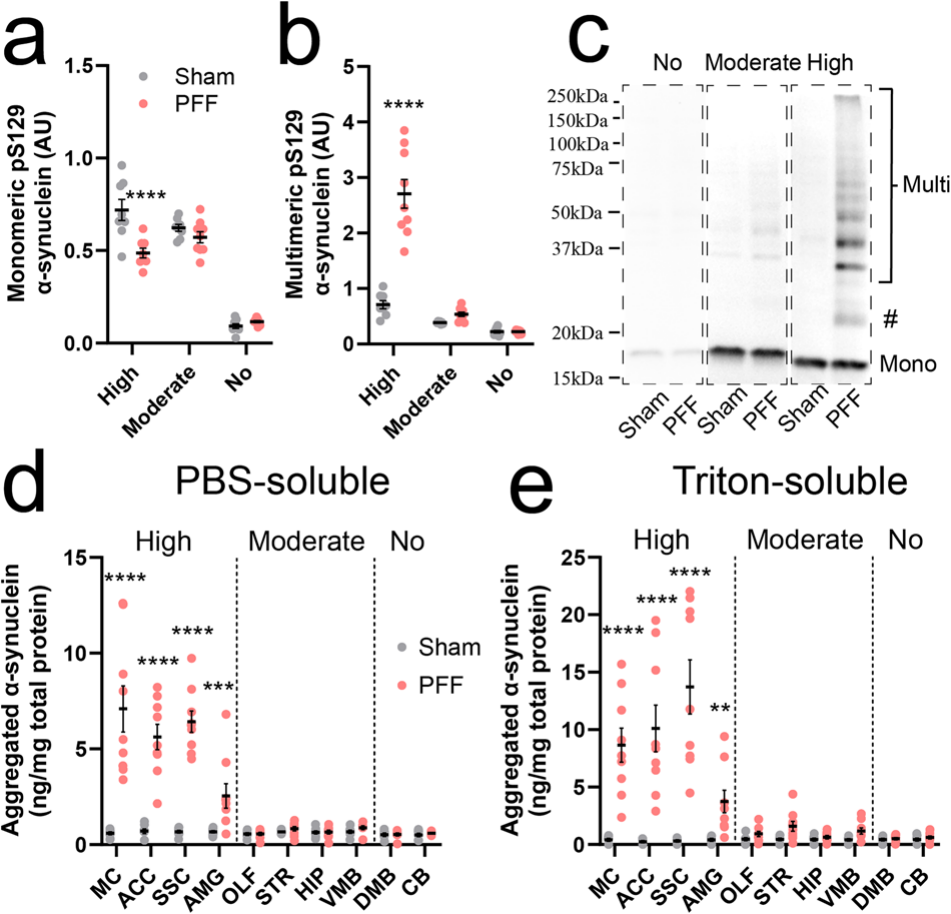
Profiling α-synuclein aggregation in the PFF mouse brain. Semi-quantitative analysis of monomeric (**a**) and multimeric (**b**) pS129 α-synuclein (D1R1R, Cell Signalling Technology^®^) was performed in whole tissue extracts from representative sham and PFF mouse brain regions exhibiting high (ACC), moderate (STR) or no (CB) pS129 α-synuclein pathology using immunoblotting (*n* = 8–9/group/category). **c** Representative pS129 α-synuclein immunoblots from each representative brain region (ACC, STR, CB; full blots displayed in Supplementary Fig. 8). All regions exhibited monomeric (Mono) pS129 α-synuclein, while multimers (Multi) were also observed in regions of high and moderate pS129 α-synuclein burden. pS129 α-synuclein immunoreactivity was also observed just above the 20 kDa molecular weight marker, which is consistent with glycosylated (O-GlcNAc) monomeric α-synuclein (#). Aggregated α-synuclein was quantified in PBS- (**d**) and Triton-soluble (**e**) tissue fractions from all 10 regions of the sham (*n* = 8) and PFF (*n* = 9) mouse brain using the LEGEND MAX α-Synuclein Aggregate ELISA assay. Associated data are contained in Supplementary Table 13. Datapoints in (**a**, **b**, **d**, **e**) represent the mean analyte quantity for a given sample, which was calculated from triplicate measurements of that sample in the same assay plate or across different immunoblots. Error bars in (**a**, **b**, **d**, **e**) represent mean ± standard error of the mean for that sample group. Data in (**a**) and (**b**) were generated from triplicate measurements of target analytes across three independent experiments (*N*), while those in (**d**) and (**e**) were generated from one independent experiment (*N*) performed in triplicate. **p < 0.01, ***p < 0.001, ****p < 0.0001; two-way ANOVA with Sidak’s multiple comparisons post-hoc tests, complete details of statistical tests are presented in Supplementary Table 8. ACC anterior cingulate cortex, AMG amygdala, AU arbitrary units, CB cerebellum, DMB dorso-medial midbrain, HIP hippocampus, MC motor cortex, OLF olfactory bulb, SSC somatosensory cortex, STR striatum, VMB ventral midbrain.

Aggregation of α-synuclein precedes S129 phosphorylation in PFF-inoculated mice^29^. We sought to develop new insights into the relationship between α-synuclein aggregation and S129 phosphorylation by profiling aggregated α-synuclein in the same tissue extracts as those used to measure total and pS129 α-synuclein. We first employed immunoblotting to assess alterations to the abundance of monomeric (Fig. 6a) and SDS-resistant multimeric pS129 α-synuclein species (Fig. 6b) in whole tissue extracts from PFF and sham mice (Fig. 6c). Brain regions exhibiting a high pathological burden in PFF mice (Fig. 4) exhibited a significant reduction in monomeric pS129 α-synuclein compared with corresponding sham mouse regions, which was accompanied by a significant increase in multimeric pS129 α-synuclein. These changes were diminished in brain regions exhibiting moderate pS129 α-synuclein pathology and were absent in those lacking synucleinopathy.

Next, we employed the LEGEND MAX α-Synuclein Aggregate ELISA (#448807, BioLegend^®^) to quantify the abundance of aggregated α-synuclein in PBS- (Fig. 6d) and TrX-soluble (Fig. 6e) tissue extracts from PFF and sham mice. Quantification was not performed in SDS-soluble tissue fractions due to the incompatibility of SDS with this assay. Characterization of aggregate assay performance using a purified aggregated α-synuclein standard provided with the ELISA kit revealed a LoD of 0.042 ng/mL in PBS-soluble buffer and 0.069 ng/mL in TrX-soluble buffer, with corresponding lower limits of quantification in these buffers slightly higher at 0.085 and 0.145 ng/mL, respectively (Supplementary Fig. 6). No α-synuclein aggregation was detected above the assay’s LLoQ in 95% of samples from sham mice, with low (0.9–1.9 ng/mg total protein) aggregation detected in the remaining 8-of-160 fractions from all investigated brain regions. While data were similar for PFF mouse brain regions lacking pS129 α-synuclein pathology (all below the LLoQ), brain regions exhibiting a high pS129 α-synuclein burden contained up to 20.15 ng aggregated α-synuclein per mg of total protein, which was marginally more abundant in TrX fractions (54.1–64.4% aggregates) compared with PBS fractions. Regions of moderate pS129 α-synuclein pathology contained much lower quantities of aggregated α-synuclein (1.62–2.90 ng/mg total protein). No correlations were identified between the levels of aggregated and pS129 α-synuclein in PBS- or TrX-soluble fractions from PFF mice (Supplementary Fig. 7).

## Discussion

Phospho-S129 α-synuclein is highly enriched in Lewy pathology^30^, hence technologies capable of high-throughput measurement of pS129 α-synuclein are of great interest in synucleinopathy research. Several assays have been developed to measure human pS129 α-synuclein in human or transgenic mouse tissue extracts and biofluids^12,31–36^, however many of these cannot detect mouse pS129 α-synuclein or exhibit poor sensitivity for this target, presumably owing to discrepancies in amino acid residues at 7 sites across the protein^37^. This constitutes a critical unmet need given measurement of pS129 α-synuclein in non-transgenic mouse models is key to understanding the physiological function of α-synuclein and its etiological contribution to synucleinopathies. By reformulating existing SureFire Ultra pS129 and total α-synuclein assays, we developed new ultrasensitive immunoassay technologies to quantify both mouse and human α-synuclein isoforms in mouse and human tissue and cell extracts. These assays are already commercially available (pS129, #ALSU-PASYN-B; total, #ALSU-TASYN-B; Revvity) and are designed as no-wash assays that can be completed within 2 h of incubation time using as little as 1 µL brain tissue extract or 4 µL cell lysate (10× homogenization buffer volume (µL)/tissue weight (mg)) per triplicate pS129 and total α-synuclein measurement. Their calibrator matrix (assay buffer) is also highly suitable for measuring mouse and human pS129 and WT α-synuclein in mouse and human tissue and cell extracts, provided samples have been diluted beyond their MRDs. Exhibiting a significantly higher sensitivity (5–20-fold) and wider LDR (5–10-fold) compared with alternative human α-synuclein measurement technologies^12,34,38^, these assays are ideal for accurate and high-throughput sample processing using minimal material.

Application of reformulated SureFire Ultra assays to PFF and sham mouse brain tissues in this study clearly demonstrates their potential to parse valuable information from synucleinopathy disease models. The model of intrastriatal injection of α-synuclein PFF in WT mice has become a mainstay in synucleinopathy research, recapitulating region-specific α-synuclein phosphorylation and aggregation observed in human disease^19^. It is unclear why select brain regions develop significant α-synuclein pathology above others and what role S129 phosphorylation plays in this process, largely as we have been unable to quantify mouse pS129 α-synuclein in these models in the past. While immunohistochemical characterization of pS129 synucleinopathy in PFF mice allows visualization and semi-quantitative estimation of relative pS129 α-synuclein burden across different brain regions, it cannot provide pS129 or total α-synuclein concentrations nor inform on the proportion of α-synuclein S129 phosphorylation across different brain regions. In this study, we demonstrate that these measurements are now made possible in a range of different extraction buffers using reformulated SureFire Ultra α-synuclein assays, which match closely with qualitative immunohistochemical estimates of pS129 α-synuclein pathology across the ten brain regions of interest. Regions of high, moderate and no pS129 α-synuclein immunofluorescence identified in fixed PFF mouse brain tissues can be clearly delineated using assay readouts in fresh brain tissue extracts (Fig. 4), with sham and PFF mice also differentiated by the concentration and proportion of pS129 α-synuclein in regions of high pS129 α-synuclein burden. Importantly, the comparable sensitivity of the total and pS129 assay for mouse and human recombinant α-synuclein isoforms indicates they may also be employed to measure these analytes in commonplace transgenic human α-synuclein mouse models, including M83 mice, or mice inoculated with human α-synuclein PFFs. This will, however, depend on whether separate quantification of the two species of α-synuclein needs to be performed in order to address study aims, which will not be possible using these assays.

In addition to measurement of soluble WT and pS129 α-synuclein, the quantification of aggregated α-synuclein constitutes an important metric of synucleinopathy burden in tissues and biofluids from patients and animal models. SureFire Ultra activation and lysis buffers contain several different types of detergents (SDS, Triton X-100, Tween-20), hence any aggregates or fibrils placed into these assays will be modified during the analytical process. It is for this reason that neither purified WT and pS129 α-synuclein PFFs, nor aggregates, were employed during total and pS129 assay screening and performance evaluation in this study. As a solution to this incompatibility, application of the same brain extracts to the BioLegend^®^ α-synuclein aggregate ELISA allowed us to demonstrate that the regions with the highest increases in pS129 α-synuclein burden also displayed large increases in both cytosolic and membrane-bound α-synuclein aggregates (Fig. 6e, f). This aggregation occurred alongside reductions in soluble pS129 α-synuclein, suggestive of intricate relationships between α-synuclein solubility, membrane binding, pS129 phosphorylation and conversion into SDS-insoluble aggregates. Overall, our data not only showcase the value of our pS129- and aggregate-assays in dissecting disease mechanisms in murine models, but also signify their potential in assessing the efficacy of treatments targeting α-synuclein phosphorylation or aggregation in pre-clinical murine models of synucleinopathy.

Aside from evaluating the performance of the new assays in mouse brain tissues, our data reveals novel biological insights into α-synuclein biology across the mouse brain and how this may influence the regional development of synucleinopathy upon PFF inoculation. Remarkable consistency in % α-synuclein S129 phosphorylation across most mouse brain regions in the WT mice highlights tight regulation of this modification under physiological conditions^21,39,40^. It is unclear whether its selective enrichment within the olfactory bulb signposts a differential regulation in this region. Others have suggested that the higher baseline pS129 α-synuclein point to the olfactory bulb as a regional nucleation point for the spread of synucleinopathy throughout the brain^20^, however, our data indicate that this would have to occur in the absence of significant increases in total, proportional pS129 α-synuclein or aggregated α-synuclein in this region (Figs. 4c, e and 6e, f). Detailed examination of α-synuclein pathology across the PFF mouse brain at multiple ages post-inoculation is needed to comprehensively address this point. It must also be acknowledged that, while PFF mice are a suitable model for studying the development of synucleinopathy, they do not necessarily recapitulate the natural regional expression of this pathology across all human synucleinopathies alike, irrelevant of injection site.

A plausible interpretation of our data is that higher baseline total and pS129 α-synuclein concentrations in certain brain regions may underlie their susceptibility to developing moderate-to-severe synucleinopathy upon intrastriatal PFF inoculation. Indeed, increased concentrations of α-synuclein enhance the kinetics of fibrillization^41^, as a corollary, the development of synucleinopathy throughout the brain may be more dependent on regional α-synuclein concentrations rather than bio-distribution from the injection site. It is interesting, therefore, that we also observe reductions in total α-synuclein in these regions of the PFF mouse brain in this study, although results were not cross-validated using alternative techniques due to limited sample availability and must therefore be interpreted with caution. Further investigations into the development of synucleinopathy in mice following varied anatomical inoculations from the same PFF batch are warranted to assess the validity of this theory.

In addition to α-synuclein concentration, our data highlight relationships between the distribution of α-synuclein among PBS-, TrX- and SDS tissue fractions and synucleinopathy load following PFF inoculation, with higher baseline levels of TrX-soluble α-synuclein predisposing select brain regions to developing more severe synucleinopathy in PFF mice. One possible explanation for this observation is that TrX-soluble α-synuclein largely constitutes forms of the protein that are membrane-bound endogenously. Under physiological conditions, α-synuclein is thought to exist in a dynamic equilibrium between its natively unfolded cytosolic isoform and membrane-bound species^42,43^, with the latter exhibiting a high aggregation propensity that can seed the aggregation of cytosolic α-synuclein^44^. The selective enrichment of membrane-bound proteins in TrX-soluble tissue fractions has been shown previously by our group^28^ and others^45,46^, although no proteomic analyses were conducted on tissue fractions in this study to confirm such a distribution. Existing estimates of the proportion of membrane-bound α-synuclein come largely from application of semi-quantitative techniques to brain tissue extracts generated using different fractionation methods that to employed in this study. For example, immunoblot analyses of α-synuclein compartmentalization in whole rat brain extracts subjected to a 5-step fractionation protocol estimated that up to 15% of α-synuclein is bound to membranes^44^. While this is significantly higher than our estimates of this percentage in the CB and DMB, it is closer to our estimates of this percentage in cortical and limbic regions (up to 7.5%); a difference which we attribute both to the poorer sensitivity of immunoblotting, together with the different fractionation protocol employed. Nonetheless, these data support our hypothesis that heterogeneity in the proportion of α-synuclein in different fractions may constitute an important determinant of the pattern of α-synuclein pathological development in PFF mice.

In summary, we reformulated existing SureFire Ultra pS129 and total α-synuclein assays to enable quantification of both mouse and human α-synuclein isoforms in mouse and human tissue and cell extracts with extremely high sensitivity. By combining these assays with an aggregate α-synuclein ELISA, we highlight the ability of these assays to leverage novel biological insights into α-synuclein biology from established synucleinopathy mouse models by enabling quantification of both pS129 α-synuclein and aggregate pathology in these models. Application of these assays to PFF and sham mouse brain tissue fractions provided quantitative estimates of absolute, proportional pS129 α-synuclein and aggregate concentrations across the mouse brain under physiological and pathological conditions. These data support a model where higher baseline concentrations of membrane-bound α-synuclein in select brain regions may promote aggregation and phosphorylation of the protein at serine residue 129, although further studies are needed to clarify the order of these molecular events. Future application of these new technologies to pre-clinical murine models of synucleinopathy employed in drug discovery studies also has the potential to improve outcome monitoring for therapies targeting pS129 and non-modified α-synuclein.

## Methods

### The AlphaLISA SureFire Ultra platform

For the purposes of this project, we obtained complete AlphaLISA SureFire Ultra kits containing all assay components as preformulated ready-to-use solutions. This not only minimized assay complexity, improved robustness and enhanced reproducibility, but more importantly will enable other investigators to obtain the same material from the supplier directly and perform their experiments as described herein. As detailed below, our work included assessment of multiple new formulations of SureFire Ultra Total α-synuclein and Phospho-α-synuclein (Ser129) kits, as well as previous formulations of these kits (PerkinElmer, Total α-synuclein; ALSU-TASYN-A, Phospho-α-synuclein (Ser129); ALSU-PASYN-A). What is described in this section applies to all tests that have been performed as well as the final product that has since been released by the manufacturer (Revvity, Total α-synuclein; ALSU-TASYN-B, Phospho-α-synuclein (Ser129); ALSU-PASYN-B).

The AlphaLISA SureFire Ultra is a high sensitivity, high-throughput assay for the robust detection of analytes in biological samples. It is a no-wash immunoassay whose function is based on the luminescent proximity principal using oxygen-channeling chemistry, as shown in Supplementary Fig. 1. In brief, its design utilizes two antibodies against a target protein, one of which is biotinylated and the other of which is conjugated to a proprietary CaptSure™ tag. The differential tagging of these antibodies enables them to selectively bind to one of two types of Alpha beads; Alpha donor beads are streptavidin-coated to allow capture of the biotinylated antibody, whilst Alpha acceptor beads are coated with a proprietary CaptSure™ agent to allow binding of the respectively tagged antibody. Once all components have been added to the sample and both antibodies and beads are complexed with the target protein, the sample is illuminated using 680 nm light, causing the release of singlet oxygen molecules from the donor beads, triggering the acceptor beads in close proximity (<200 nm) to emit a signal at 615 nm. This emission intensity is directly proportional to the amount of the target protein within the assay’s dynamic range.

The assay was performed according to the manufacturer’s recommended protocol, with the exception that the positive control lysate supplied with each kit was replaced by purified human or mouse WT α-synuclein, or pS129 α-synuclein protein standards, which are described below. Assay signals were measured using an EnVision™ multimodal plate reader under the manufacturer’s preset AlphaScreen™ protocol.

### Recombinant α-synuclein protein standards

Mouse (#RP-009) and human (#RP-003) WT α-synuclein, as well as human pS129 α-synuclein (#RP-004), were obtained from Proteos (Kalamazoo, MI, USA), where they were produced in collaboration with the Michael J Fox Foundation for Parkinson’s Research. Recombinant mouse and human WT α-synuclein protein standards were provided as 100 µL stock aliquots of 10 mg/mL α-synuclein in 10 mM Tris, 50 mM NaCl, pH 8, which were diluted to 1 mg/mL intermediate working aliquots using 900 µL of 10 mM Tris, 50 mM NaCl, pH 8 containing 0.55% Tween-20, 0.55% Triton X-100, 0.55% bovine serum albumin (BSA) and 0.055% sodium azide (final concentrations: 10 mM Tris, 50 mM NaCl, pH 8 containing 0.5% Tween-20, Triton X-100 and BSA, 0.05% sodium azide). Recombinant human pS129 α-synuclein was provided as 1 mg aliquots of lyophilized standard, which were reconstituted to 1 mg/mL intermediate working aliquots using 1 mL of 10 mM Tris, 50 mM NaCl, pH 8 containing 0.5% Tween-20, Triton X-100 and BSA, 0.05% sodium azide.

Mouse pS129 α-synuclein was not available from the same supplier. We, therefore, produced it in-house from a 1.25 mg/mL solution of mouse WT α-synuclein in kinase reaction buffer, which was generated by diluting a 100 µL (10 mg/mL) aliquot of this standard eightfold in 700 µL of 22.86 mM HEPES, 1.25 mM ATP, 2.29 mM DTT, 11.43 mM MgCl_2_, pH 7.4 (final concentrations: 20 mM HEPES, 1.09 mM ATP, 2 mM DTT, 10 mM MgCl_2_, pH 7.4). Phosphorylation was induced by incubating 1 µg of recombinant human PLK3 protein (Thermo Fisher Scientific, MA, USA; #PR7316B) with 50 µL of mouse WT α-synuclein in kinase reaction buffer for 8 h at 30 °C. Immunoblotting and electrospray ionization mass spectrometry were used to confirm complete phosphorylation of α-synuclein S129 (Supplementary Fig. 9). All standards were stored at −80 °C in 10 µL aliquots, with the exception of lyophilized human pS129 α-synuclein, which was stored at −20 °C as per manufacturer’s recommendations. Any unused standard leftover from a thawed aliquot was discarded at the end of each assay working day.

### HEK293 cell culture and transfection

WT (#ab255449) and α-synuclein KO (#ab255433) HEK293 cells were obtained from Abcam, while PLK3-HEK293 cells were produced by transfection of WT HEK293 cells with Myc-DDK-tagged human PLK3 (# RC203352, OriGene, MA, USA). All experimental procedures conducted on these cell lines were approved by the University of Sydney Institutional Biosafety Committee (#21E012).

For transfection experiments, plasmids were first transformed into One Shot TOP10 Chemically Competent *Escherichia coli* (Thermo Fisher Scientific; #C404003) according to manufacturer’s instructions. Selection for successful transformation was performed using Ampicillin (100 µg/mL, control) or Kanamycin (25 µg/mL, PLK3) prior to the expansion of resistant colonies in 100 mL of Luria-Bertani (LB) broth and purification of plasmid DNA using a PureLink HiPure Plasmid Filter Midiprep Kit (Thermo Fisher Scientific; #K210014), according to manufacturer’s instructions. Aliquots of transformed bacteria were stored in a 1:1 mixture with 50% glycerol at −80 °C, while purified plasmid DNA was stored reconstituted in deionized water at −20 °C. WT and α-synuclein KO HEK293 cells were then seeded into 6-well plates (45× 10^4^ cells/well) before being transfected with 5 µg DNA per well using Lipofectamine 3000 (Thermo Fisher Scientific; #L3000015) according to manufacturer’s instructions. Cells in were grown to 90% confluency before being harvested, washed in 1x PBS, pelleted, and stored until use at −80 °C.

For assay validation experiments, transfected and non-transfected cells were grown to 90% confluency in 6-well plates using a 1:1 mixture of Dulbecco’s Modified Eagle Medium and Nutrient Mixture F-12 (DMEM/F-12, Thermo Fisher Scientific; #11320033) supplemented with 10% fetal bovine serum (heat inactivated, Thermo Fisher Scientific; #16000044), 1% GlutaMAX (Thermo Fisher Scientific; #35050061) and 1% penicillin–streptomycin (10,000 U/mL, Thermo Fisher Scientific; #15140122), before being harvested, washed in 1x PBS and pelleted. Cell pellets were resuspended in 1x assay lysis buffer containing protease (Sigma; #11697498001) and phosphatase (Sigma; #4906845001) inhibitors (10 volumes (µL) per mg cell pellet) using a Branson SFX250 Sonifier (Emerson, St. Louis, MO, USA; #101-063-965R) equipped with a 3/32″ probe microtip. Pellets were sonicated on ice for 2 min with a 5 s pulse (70% amplitude), followed by a 25 s pause.

### Wild-type and α-synuclein knock-out mice

Female WT C57BL/6 mice (12 weeks old) were obtained from the Animal Resources Centre (Canning Vale, WA, Australia) and housed within the Laboratory Animal Services facility at the Charles Perkins Centre (University of Sydney, NSW, Australia), with ad libitum access to food and water on a 12 h light–dark cycle at temperatures between 20 and 24 °C and humidity between 40 and 70%. All experimental procedures were approved by the University of Sydney Animal Ethics Committee (#2021/2015).

Brain tissues were excised from WT and α-synuclein KO mice following intraperitoneal injection with a lethal dose of sodium pentobarbitone (Lethabarb, 100 mg/kg) and transcardial perfusion with ice-cold 0.9% NaCl for 10 mins at a constant flow rate of 6 mL/min using a peristaltic pump (World Precision Instruments, Sarasota, FL, USA). Brains were then sagittally bisected, with one hemisphere frozen immediately on dry ice and stored at −80 °C. The contralateral hemisphere was cut into four pieces of approximately equal size, which were then sonicated for 3 min or until no visible tissue pieces remained using the same microtip-based sonication protocol described above for cell extracts. These four homogenates were then combined to create a whole brain homogenate, which was centrifuged at 20,000 × *g* for 30 min at 4 °C before the supernatant was collected and aliquoted for subsequent analyses.

Homozygous *Snca* knock-out mice were obtained from Jackson Laboratories (SNCA^−/−^; C57BL/6N-Snca^tm1Mjff^/J; JAX stock #016123)^47^. They were bred under the breeding permit 2022-15-0202-001135 issued by the Danish Veterinary and Food Administration. Animals were housed with light–dark cycles of 12 h intervals. Mice were fed standard chow and water ad libitum. Brain tissues were excised from 12-week-old WT and α-synuclein KO mice following intraperitoneal injection with a lethal dose of sodium pentobarbitone (Lethabarb, 100 mg/kg) and transcardial perfusion with ice-cold 0.9% NaCl for 10 min at a constant flow rate of 6 mL/min using a peristaltic pump (World Precision Instruments, Sarasota, FL, USA). Upon removal, brains were dissected sagittally down the midline and snap-frozen in liquid nitrogen.

### SureFire assay validation

Five new total and pS129 assay antibody pairings were screened against purified mouse and human WT and pS129 α-synuclein protein standards, which we named as total or pS129 α-synuclein assay pair 1-5 in this publication. Antibody pairs were all designed to contain at least one antibody known to robustly detect mouse and human α-synuclein in fixed and/or frozen tissue sections and protein extracts using immunohistochemistry and/or immunoblotting. Standards were prepared for assay screening by serially diluting 1 mg/mL intermediate working aliquots of each standard to concentrations between 2.24 ng/mL and 0.038 pg/mL using 1x Alpha SureFire Ultra Lysis Buffer (Revvity, MA, USA; #ALSU-LB-100ML), hereon referred to simply as assay lysis buffer. Serial dilutions for a given standard were all performed using the same pipette tip and were conducted in Protein LoBind^®^ Tubes (Eppendorf, Hamburg, Germany; #0030108434) to minimize loss of α-synuclein between dilutions through adherence to pipette tips and tube walls. Each dilution of every standard was measured in three technical replicates and data were expressed as mean ± standard deviation to convey average assay signal and intra-assay variation. We defined the LoD and LLoQ as 3 and 6 standard deviations above the mean of the blank, respectively. Inter-assay variability for each dilution of every standard was determined across three individual standard curves measured on separate days and was calculated by dividing the standard deviation of the replicates by their mean, multiplied by 100 to produce percentage variability.

The 2 formulations of each assay with the highest sensitivity for mouse and human isoforms of their target protein were then applied to serially diluted WT and α-synuclein KO mouse brain tissue supernatant and HEK293 cell lysates, as well as PLK3-HEK293 cell lysates. This was undertaken to assess whether the assay exhibiting the greatest sensitivity for purified protein standards was also the formulation with the lowest amount of interference from additional sample components present in tissue or cell lysates—known as matrix effects. Extracts from WT mouse brain, HEK293 cells and PLK3-HEK293 cells were used to evaluate the comparative sensitivities of assay formulations for α-synuclein in a complex sample matrix, while KO extracts were used to assess whether false-positive assay signals were being generated from non-specific interactions between assay reagents and extract components other than α-synuclein. Each dilution of every sample was measured in three technical replicates and assay data (intra- and inter-assay variability, LoD, LLoQ) calculated as described for purified standards above. All subsequent experiments only employed the total and pS129 α-synuclein assay formulation exhibiting the highest sensitivity.

For parallelism experiments, WT and α-synuclein KO mouse brain tissue extracts were each pre-diluted 10- and 100-fold in assay lysis buffer, representing dilution factors above and below the MRD for mouse brain matrix. These pre-diluted WT extracts were then serially diluted up to 4096-fold using either assay lysis buffer or the equivalent dilution of α-synuclein KO extract, i.e. 10-fold diluted WT extract was diluted up to 4096-fold using 10-fold diluted KO extract. WT and α-synuclein KO HEK293 cell lysates were processed in a similar manner, being pre-diluted 5- and 20-fold in assay lysis buffer.

Spike-in experiments were designed to assess recovery of WT and pS129 mouse α-synuclein from mouse brain tissue matrix. WT mouse brain tissue extract was diluted 2000-fold in assay lysis buffer to ensure signal from endogenous α-synuclein was within the assay’s LDR, whereas α-synuclein KO extract was diluted 100-fold in assay lysis buffer to ensure minimal signal distortion by non-specific binding to matrix components (MRD—24-fold). WT and pS129 mouse α-synuclein standards were then spiked separately into diluted WT and KO mouse brain extracts to final estimated concentrations ranging between 10–640 pg/mL (WT protein) and 0.625–40 pg/mL (pS129 protein). These concentration ranges were designed to fall within the LDR of the total and pS129 α-synuclein assay, respectively. WT mouse α-synuclein spike recovery was assessed with the total assay and pS129 mouse α-synuclein spike recovery was assessed with the pS129 assay. Measured spike concentration was calculated using the increase in assay signal above baseline signal generated by assay buffer, WT extract or KO extract, which was then expressed as a % of the intended spike concentration (% recovery). Measured spike concentrations for WT mouse α-synuclein were determined using the total α-synuclein kit standard curve for WT mouse α-synuclein, while those for pS129 mouse α-synuclein were determined using the pS129 α-synuclein kit standard curve for pS129 mouse α-synuclein.

### Mouse α-synuclein pre-formed fibril (PFF) generation and characterization

WT monomeric mouse α-synuclein was produced as previously described^48^. Lyophilized WT monomeric α-synuclein was reconstituted in PBS pH 7.4 (Gibco, Billings, MT, USA) and passed through a 100 kDa Amicon^®^ Ultra Centrifugal Filter (Merck, Rahway, NJ, USA) to separate unwarranted oligomeric species, before being sterile filtered through a 0.22 µm filter (Merck) to remove additional particulates and microorganisms. Protein concentration was determined using a Pierce™ BCA Protein Assay Kit (Thermo Fisher Scientific™) according to manufacturer’s instructions and adjusted to 1.025 mg/mL with PBS. Next, 25 µL of 2 mg/mL sonicated α-synuclein PFFs (produced previously^49^) was added to 975 µL of the 1.025 mg/mL monomeric α-synuclein solution to seed aggregation, at a concentration of 5% fibrils by mass%. The samples were incubated at 37 °C for 72 h on an orbital shaker (1050 rpm). Samples were centrifuged at 15,600 × *g* for 30 min to pellet insoluble fibrils, which were subsequently resuspended in PBS. The protein concentration in this suspension was again determined using a Pierce™ BCA Protein Assay (Thermo Fisher Scientific) and adjusted to 2 mg/mL using PBS. Fibrils were then sonicated for 20 min with 30 ms pulses at 30% power, followed by 70 ms pauses, using a Branson SFX250 Sonifier equipped with a 1″ cup horn (Branson; #101-147-046), before aliquots were stored at −80 °C. Amyloid structure of fibrils was confirmed using a Thioflavin-T binding assay as previously described (Supplementary Fig. 10)^50^, while fibril fragment size (24 nm) was measured by dynamic light scattering using a DynaPro™ NanoStar™ (Wyatt Technology, Goleta, CA, USA) and was not found to change following freeze/thaw (Supplementary Fig. 10). Fibrils were also subjected to sedimentation analysis, whereby sonicated PFFs and monomeric standards were centrifuged at 25,000 × *g* for 30 min. Both the pellet and supernatant were then collected, the pellet resuspended in PBS, and immunoblot loading buffer was added to both fractions. Samples were then incubated at 95 °C for 10 min, before being loaded into pre-cast gels and subjected to electrophoresis as described above. Proteins were stained within gels using Coomassie Blue and imaged using a Fuj LAS-3000 Intelligent Dark Box (Fujifilm, Japan). Approximately 50% of the PFFs became soluble after sonication and freeze/thaw (Supplementary Fig. 10), which we attributed to small fragment size given dynamic light scattering data did not indicate monomerization following freeze/thaw.

### Wild-type mouse stereotaxic injections

C57BL/6J mice weighing ~20 g were given prophylactic Temgesic (0.03 mg/kg) intraperitoneally 15 min prior to anesthesia induction with inhalable isoflurane (2–5%). Once anesthesia was achieved, the dorsal head surface was shaved and animals were secured to the stereotaxic frame (Stoelting, Wood Dale, IL, USA), fitted with a mouse face mask adapter. A 5 mm midline incision was made on the dorsal surface, the tissue retracted and bregma located. Animals received bilateral 2.5 µL intracerebral injections through a drilled burrhole of either 2 mg/mL mouse α-synuclein PFF’s (PFF; *n* = 12) or sterile PBS (pH 7.4, sham; *n* = 9) targeting the center of the STR (relative to bregma: +0.2 mm anteroposterior, ±2 mm mediolateral, −3.2 mm dorsoventral from the dural surface). Injections were performed with a glass cannula (50 μm internal diameter) attached to a 10 μL Hamilton syringe (Hamilton Company, NV, USA) and injected at a constant dose rate of 0.4 μL/min using an automated syringe pump (UltraMicroPump3, World Precision Instruments, FL, USA). The cannula was kept in-situ for 5 min at each target site prior to retraction and flushing with 0.9% sterile saline between injections. Following surgery, skin incisions were closed with suture and animals monitored closely during recovery.

### PFF mouse brain tissue harvesting and processing

Sham and PFF mice (*n* = 3/group) culled for histological profiling of pS129 α-synuclein burden 3 months post-intrastriatal PFF inoculation were injected intraperitoneally with a lethal dose of sodium pentobarbitone (Lethabarb^®^, 200 mg/kg), before being transcardially perfused with 0.9% NaCl (8 min, 6 mL/min) followed by ice-cold 4% paraformaldehyde (PFA in 0.1 M phosphate buffer, pH 7.4, 8 min, 6 mL/min) using a peristaltic pump (World Precision Instruments). Brains were excised whole, post-fixed in 4% PFA overnight at 4 °C and transferred into 30% sucrose (w/v in 1x PBS) for 48 h at 4 °C. Brains were then flash frozen using isopentane cooled to between −50 °C and −60 °C on dry ice, before being mounted onto microtome chucks using optimal cutting temperature compound. Whole mounted brains were sectioned coronally into 12 × 30 µm section series’ and stored in antifreeze solution (30% glycerol, 30% ethylene glycol in 1x phosphate buffer (PB; pH 7.4)) at −20 °C.

Sham and PFF mice (*n* = 8–9/group) culled for biochemical analyses 3.5 months post-intrastriatal PFF inoculation were lethally anaesthetized and transcardially perfused with 0.9% NaCl as above, before brains were harvested whole and bisected along the mid-sagittal axis. The right hemisphere was post-fixed in 4% PFA overnight at 4 °C, transferred into 70% ethanol overnight at 4 °C, and embedded in paraffin wax. The left hemisphere was dissected fresh to obtain tissue from 10 brain regions—the OLF, MC, ACC, SSC, AMG, STR, HIP, VMB, CB and DMB. These specific brain regions were chosen for further biochemical examination as they exhibited high (MC, ACC, SSC, AMG), mild-moderate (OLF, STR, MB, HIP) or no (CB, DMB) pS129 α-synuclein burden in histological analyses of PFF brains at 3 months post-PFF inoculation.

Fresh tissues were suspended in 1x assay lysis buffer containing protease (Sigma; #11697498001) and phosphatase (Sigma; #4906845001) inhibitors (10 volumes (µL) per mg tissue) using a Branson SFX250 Sonifier (Emerson, St. Louis, MO, USA; #101-063-965R) equipped with a 3/32″ probe microtip. Pellets were sonicated on ice for 2 min with a 5 s pulse (70% amplitude), followed by a 25 s pause. One quarter of the total homogenate volume was then aliquoted and stored at −80 °C (“whole tissue extract”), while the remainder was centrifuged at 20,000 × *g* for 30 min at 4 °C and the supernatant collected and designated the “PBS-soluble fraction”. PBS-insoluble pellets were then resuspended in 10 volumes (µL) of PBS fraction buffer supplemented with 0.5% Tween-20 and 0.5% Triton X-100 (TrX) using probe tip sonication (2× 5 s pulses, 70% amplitude, 25 s intermittent pause) and incubated on ice for 1 h. These solutions were then centrifuged at 20,000 × *g* for 30 min at 4 °C and the supernatant collected and designated the “TrX-soluble fraction”. Any remaining TrX-insoluble pellets were fully dissolved in TrX fraction buffer supplemented with 2% SDS using probe tip sonication as described for the TrX fraction, and were designated the “SDS-soluble fraction”. Protein concentrations in PBS, TrX and SDS fractions were determined using a Pierce™ BCA Protein Assay (Thermo Fisher Scientific). The robustness of this fractionation protocol is evidenced by the very consistent proportion of total and pS129 α-synuclein in the three fractions generated from each sham mouse brain region (Supplementary Fig. 11), with greater variability in PFF mice likely due to the biological impact of PFF inoculation. Any cross-contamination between fractions would have manifested as a transfer of material from the PBS fraction into other fractions, however this is highly unlikely to have occurred given that almost all pS129 α-synuclein was PBS-soluble in sham mice.

### Immunofluorescence

Fixed tissue sections from sham and PFF mice were prepared for fluorescent staining and microscopy by first washing in PBS containing 0.1% Tween-20 (PBS-T) to remove antifreeze solution, before being subjected to antigen retrieval in citrate buffer (10 mM sodium citrate, 0.05% Tween-20, pH 6.0) at 70 °C for 30 min. Sections were then washed again with PBS-T before being blocked (4% BSA (w/v), 1% casein (w/v), 1.5% glycine (w/v), 0.25% Triton X-100 (v/v) in 1x PBS) for 1 h at room temperature and incubated overnight at 4 °C with primary antibodies directed against pS129 α-synuclein (rabbit, 1:10,000, EP1536Y, Abcam; #ab51253), tyrosine hydroxylase (chicken, 1:5,000, Abcam; #ab76442) and NeuN (rat, EPR12763, Abcam; #ab279297) diluted in blocking serum. Primary antibodies were detected using goat anti-host IgG secondary antibodies conjugated to AlexaFluor™ 405, 488 or 647 dyes (1:1000 diluted in blocking solution), before sections were mounted onto microscope slides coated with gelatin-chrom alum and coverslipped using #1.5 coverslips and ProLong™ Diamond Antifade Mountant (Thermo Fisher Scientific). Images were collected using a C2 laser scanning confocal microscope equipped with 405, 488 and 640 laser lines (Nikon, Minato-ku, Tokyo, Japan), as well as a Nikon Plan Apo VC 20x (0.75 DIC N2, 0.17 WD 1.0) and a Nikon CFI Plan Apochromat Lambda D 40x objective (0.95 DIC N2, 0.21 WD 1.0), Images were viewed and analyzed using Fiji software (National Institute of Health, Bethesda, MD, USA).

### SureFire assay measurements in PFF mouse brain extracts

We first calculated the optimal dilution factors required to bring total and pS129 α-synuclein levels in each synucleinopathy mouse model, extract, and/or fraction, to within the LDRs of the total and pS129 assay (Supplementary Table 14). These calculations also considered the magnitude of dilution required to reduce the concentrations of detergents present in some sample buffers (TrX and SDS) below their maximum compatible concentration (data available from Revvity website). Revvity previously determined that TrX concentrations up to 3% do not negatively impact assay signal, while 0.07% SDS causes <10% dampening of assay signal, which increases to 50% signal dampening at 0.1% SDS. We cross-validated these findings by producing 1%, 0.1% and 0.01% solutions of PBS, TrX and SDS in 1x assay lysis buffer, and then serially diluted WT and pS129 α-synuclein standards in these various buffers, before measuring their assay signal (Supplementary Fig. 12). In this design, any buffer interferences would be reflected as rightwards shifts in the dilution curves of these standards. We determined that PBS and TrX did not impact the performance of the total or pS129 assay at any concentration tested, hence we can be confident that PBS and TrX fraction buffers did not interfere with total or pS129 α-synuclein measurements. By contrast, SDS significantly dampened assay signal at concentrations between 1 and 0.1%, consistent with data presented by Revvity. Given our SDS fraction buffer contains 2% SDS, this means we must dilute this buffer >20-fold to mitigate any interference, which is why SDS-fraction samples were diluted 50-fold prior to total or pS129 α-synuclein measurements.

Following identification of optimal dilution factors for whole tissue extracts (homogenates), as well as PBS-, TrX- and SDS-soluble brain tissue fractions, samples were then diluted in assay lysis buffer to their optimal dilution factors and total and pS129 α-synuclein were measured. All dilution factors were beyond the MRD (24-fold) required to alleviate mouse brain matrix effects. Total α-synuclein concentration in diluted samples was calculated using the total α-synuclein kit standard curve for WT mouse α-synuclein. Phosphorylated S129 mouse α-synuclein was determined using the pS129 α-synuclein kit standard curve for pS129 mouse α-synuclein. Concentrations in diluted samples were then multiplied by their dilution factors and expressed as ng/mg total protein to account for any differences in protein concentration in original extracts. The proportion of α-synuclein phosphorylated at S129 was calculated by dividing ng pS129 α-synuclein/mg total protein by the ng total α-synuclein/mg total protein, expressed as a percentage.

### Immunoblotting

Immunoblotting for total and pS129 α-synuclein in protein standards (500 ng) or tissue/cell extracts (20 µg total protein) was performed by incubating samples in loading buffer (100 mM dithiothreitol (DTT), 3% SDS, 10% glycerol, 0.05% bromophenol blue, 62.5 mM Tris-base, pH 7.4; Sigma-Aldrich, MO, USA) at 95 °C for 10 min before being loaded into 4–20% Mini-PROTEAN^®^ TGX™ Precast Protein Gels (15 wells, 15 µL; Bio-Rad, CA, USA) and subject to electrophoresis on a Bio-Rad Mini-PROTEAN^®^ Tetra Cell system (180 V, 40 min, 4 °C). Proteins were then transferred to Immun-Blot^®^ PVDF membrane (0.2 µm pore size; Bio-Rad) at 30 V for 2 h at 4 °C, before membranes were fixed in 4% paraformaldehyde for 30 min at room temperature, washed in 1x PBS containing 0.1% Tween (PBS-T) and blocked in 5% BSA (Sigma-Aldrich, #A7030) diluted in PBS-T for 1 h at room temperature. Membranes were incubated overnight at 4 °C with a mouse pan α-synuclein primary antibody (Syn1, BD Biosciences, NJ, USA; #610786) diluted 1:5000 in blocking solution, before being washed with PBS-T and incubated for 2 h at room temperature with 1:5000 goat anti-mouse horseradish peroxidase-conjugated secondary antibody (Thermo Fisher Scientific; #G21040) diluted in blocking solution. Protein signals were developed using ECL western blotting substrate (Bio-Rad) and detected using a Chemi-Doc™ XRS imaging system (Bio-Rad) according to manufacturer’s instructions. Membranes were then incubated in stripping buffer (25 mM glycine, 2% SDS, pH 2.0) for 30 min at room temperature, before being washed, blocked, probed and imaged as above using a rabbit pS129 α-synuclein-specific primary antibody (D1R1R, 1:5,000, Cell Signalling, MA, USA; #23706) and a goat anti-rabbit horseradish peroxidase-conjugated secondary antibody (1:5000, Thermo Fisher Scientific; #G21234).

For quantitation of synaptophysin in mouse brain extracts, 7 µg of total protein was incubated in loading buffer as described above and loaded into AnykD Mini-PROTEAN TGX Precast Protein Gels (15 wells, 15 µL; Bio-Rad). Electrophoresis and protein transfer were performed as above, without membrane fixation, before membranes were dried overnight 2 h at room temperature and proteins stained with Sypro Ruby Protein Blot Stain (Thermo Fisher Scientific), according to the manufacturer’s instructions. Sypro Ruby-stained membranes were imaged using a Chemi-Doc™ XRS imaging system (630BP30nm filter, Bio-Rad), and total protein in each sample quantified by densitometry using ImageLab software (v.5.2, Bio-Rad). Following total protein quantification, membranes were blocked in 5% skim milk diluted in PBS-T for 1 h at room temperature and incubated overnight at 4 °C with a rabbit synaptophysin primary antibody (YE269, Abcam, Cambridge, UK; #ab32127) diluted 1:2000 in blocking solution. Membranes were then washed with PBS-T and incubated for 2 h at room temperature with 1:3000 goat anti-rabbit horseradish peroxidase-conjugated secondary antibody (Thermo Fisher Scientific; #G21234) diluted in blocking solution. Protein signals were developed and detected as above, and synaptophysin protein levels normalized to total protein levels within each sample to correct for variations in protein loading, as well as an internal standard (pooled from all tissue samples) to correct for variability between gels and immunoblot runs.

### α-Synuclein aggregate quantification

Aggregated α-synuclein was quantified in PBS- and TrX-soluble tissue fractions (5 µg total protein/well) using the LEGEND MAX™ α-Synuclein Aggregate ELISA (BioLegend) according to manufacturer’s instructions. Standard curves were constructed using the purified α-synuclein aggregate standard that is provided with the assay kit. Additional validation data regarding the specificity, sensitivity, linearity, intra- and inter-assay precision, as well as % recovery of known amounts of mouse α-synuclein aggregates spiked into a variety of sample matrices, are available in the technical disclosure statement for this product (available from BioLegend website, LEGEND MAX™ α-Synuclein Aggregate ELISA Kit).

### Statistical analyses

Statistical analyses were performed using IBM SPSS Statistics (Version 27, IBM, Armonk, NY, USA) and GraphPad Prism (Version 7.02, GraphPad, San Diego, CA, USA). Parametric tests or descriptive statistics with parametric assumptions (standard two-way and one-way ANOVA, Pearson’s *r*) were used for variables meeting the associated assumptions, with data normality assessed using either the D’Agostino-Pearson (omnibus K2) normality test or the Shapiro–Wilk (Royston) normality test. Two-way ANOVAs were employed to examine data variation across brain regions (either individually or grouped as high, moderate or no pathology) and treatments (sham vs PFF). These analyses were paired with Sidak’s multiple comparisons post-hoc tests to assess pair-wise comparisons for a given variable (e.g. a brain region of interest between sham and PFF mice, a fraction of interest between sham and PFF mice). No outliers were detected using the combined robust regression and outlier removal (ROUT) method with a maximum false discovery rate of 5%. A *p* value of <0.05 was accepted as the level of significance. Details of the statistical tests employed (test statistics, sample sizes, *p* values) for each variable of interest are included in the corresponding figure legend, results text or supplementary data section. An *n* of 8–9 cases per diagnostic group was strongly powered to detect differences in %pS129 α-synuclein (90–100% power, two-tailed *t-*test, *α* = 5%; SPSS software, IBM, Armonk, NY, USA) based on a preliminary power analysis of pilot data describing this measure in PFF- vs sham-inoculated WT mice (*n* = 3/group).

## Supplementary information

Supplementary Information

## Data Availability

All assay validation data are available in the main text or Supplementary Materials of this article. Any additional datasets generated using our experimental mouse models are available from the corresponding author on reasonable request.
