# Supplementary material for: Novel tools to quantify total, phospho-Ser129 and aggregated alpha-synuclein in the mouse brain
Source: NPJ Parkinsons Dis. 2024 Nov 8;10:217. doi: 10.1038/s41531-024-00830-y (PMC11549080; doi:10.1038/s41531-024-00830-y)
Supplement: Supplementary file 1 — Supplementary Information [file 41531_2024_830_MOESM1_ESM.pdf]

Full title: Novel tools to quantify total, phospho-Ser129 and aggregated alpha-synuclein in the mouse brain

SUPPLEMENTARY INFORMATION

Supplementary Figures 1-8

1) AlphaLISA SureFire Ultra assay platform and workflow schematics.
2) Characterization of the linear dynamic range of new SureFire Ultra assays using purified protein standards.
3) Matrix effects in HEK293 cell lysate using a parallelism experiment.
4) Immunohistochemical profiles of pS129 α-synuclein across brain regions of interest in sham and PFF mice.
5) Immunoblot quantification of synaptophysin in sham and PFF mouse brain regions.
6) Characterization of the sensitivity of the Biolegend LEGEND MAX α-Synuclein Aggregate ELISA using a purified α-synuclein aggregate standard.
7) Correlations between levels of aggregated and pS129 α-synuclein in PBS- and TrX-soluble fractions.
8) Full representative immunoblots characterising α-synuclein phosphorylation in brain tissue extracts from sham and PFF mice.
9) Full representative immunoblots characterising S129 phosphorylation in purified protein standards.
10) Characterisation of mouse pre-formed fibril (PFF) structure and solubility.
11) Proportions of total and pS129 α-synuclein in PBS, TrX and SDS fractions of sham mouse brain.
12) Evaluation of the impact of PBS, TrX and SDS on assay performance.

Supplementary Tables 1-11

1) Characterization of previous total and pS129 α-synuclein SureFire Ultra assay formulations using purified protein standards.
2) Characterization of new total and pS129 α-synuclein SureFire Ultra assay formulations using purified protein standards.
3) Characterization of the most sensitive new total and pS129 α-synuclein SureFire Ultra assay formulations using purified protein standards.
4) Characterization of new total and pS129 α-synuclein SureFire Ultra assays using extracts from mouse brain tissues and HEK293 cells.
5) Evaluation of matrix effects in mouse brain tissue extracts and HEK293 cell lysates using a parallelism experiment.
6) Evaluation of mouse brain tissue matrix effects in total α-synuclein assay using a spike-recovery experiment.
7) Evaluation of mouse brain tissue matrix effects in pS129 α-synuclein assay using a spike-recovery experiment.
8) Complete statistical test details.
9) Sham and PFF mouse brain tissue extract dilutions prior to SureFire Ultra assay measurement.

10) Percentage of total α-synuclein recovered in PBS, Triton and SDS brain tissue fractions from PFF and sham mice.
11) Percentage of pS129 α-synuclein recovered in PBS, Triton and SDS brain tissue fractions from PFF and sham mice.
12) Concentration of total α-synuclein recovered in PBS, Triton and SDS brain tissue fractions from PFF and sham mice.
13) Concentration of pS129 α-synuclein recovered in PBS, Triton and SDS brain tissue fractions from PFF and sham mice.
14) Amount of α-synuclein aggregation quantified in PBS and Triton brain tissue fractions from PFF and sham mice using the LEGEND MAX α-Synuclein Aggregate ELISA.

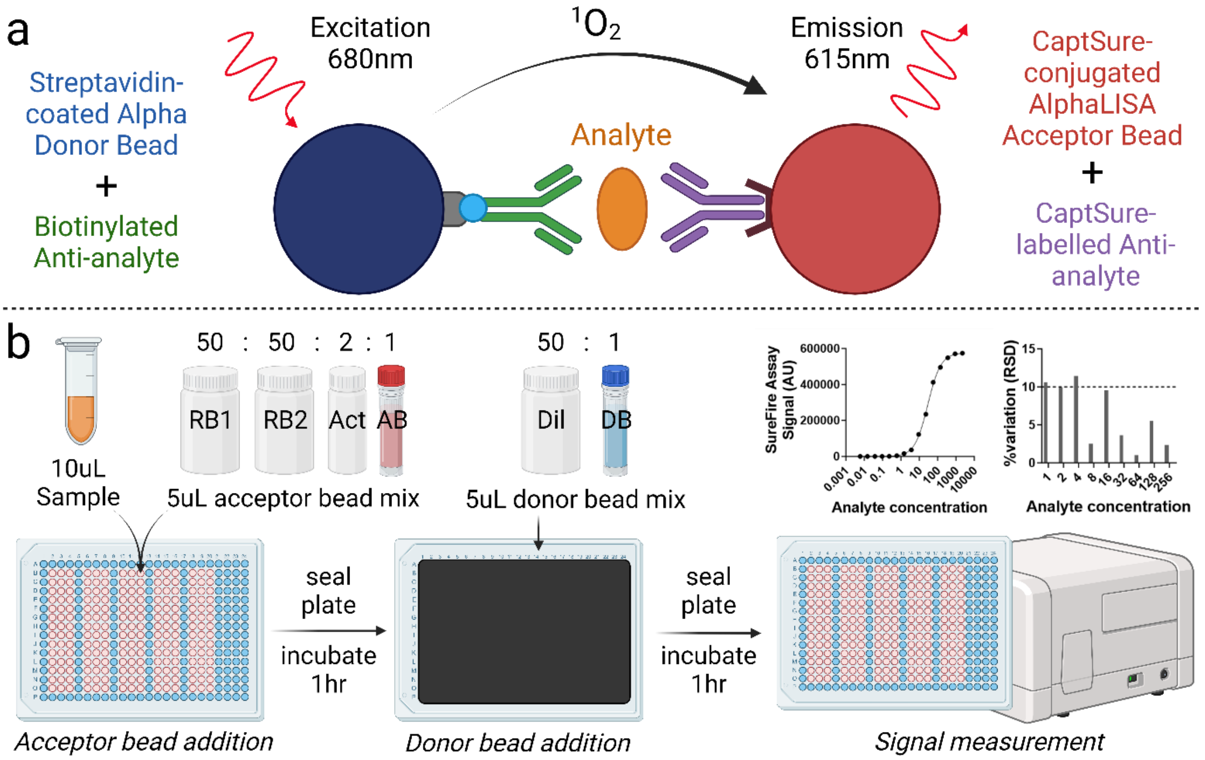

Supplementary Figure 1. AlphaLISA SureFire Ultra assay platform and workflow schematics. AlphaLISA SureFire Ultra assays are designed using two antibodies against a target protein, which are differentially tagged to ensure their selective conjugation to one of two types of Alpha beads; donor beads or acceptor beads. The binding of both antibodies to an analyte brings donor and acceptor beads into very close proximity, which enables donor beads to activate acceptor beads following sample photoexcitation to produce the assay signal (**a**). Our assay workflow followed the manufacturer's instructions (**b**). Briefly, reaction buffers 1 (RB1) and 2 (RB2) contain the assay antibodies and are combined with solutions of activation buffer (Act) and acceptor beads (AB) in a 50:50:2:1 ratio, respectively – i.e. 50µL RB1 + 50µL RB2 + 2µL Act + 1µL AB. Five microliters of this solution is added to 10µL of sample in a 384-well microtiter plate (AlphaPlate-384, Perkin Elmer, MA, USA; #6005350), which is then sealed with a light-impermeable microplate seal (TopSeal-A 384, Perkin Elmer; #6050173) and incubated for 1hr at room temperature with orbital rotation (250rpm). Next, donor beads are added to dilution buffer in a 1:50 ratio, before 5µL is added to wells and plates are again sealed and incubated for 1hr at room temperature with orbital rotation. All steps involving donors beads should be performed under subdued laboratory lighting (<100 lux), with green light filters (e.g. LEE090) recommended. Samples were then quantified using an EnVision multimodal plate reader according to manufacturer's AlphaScreen factory default protocol.

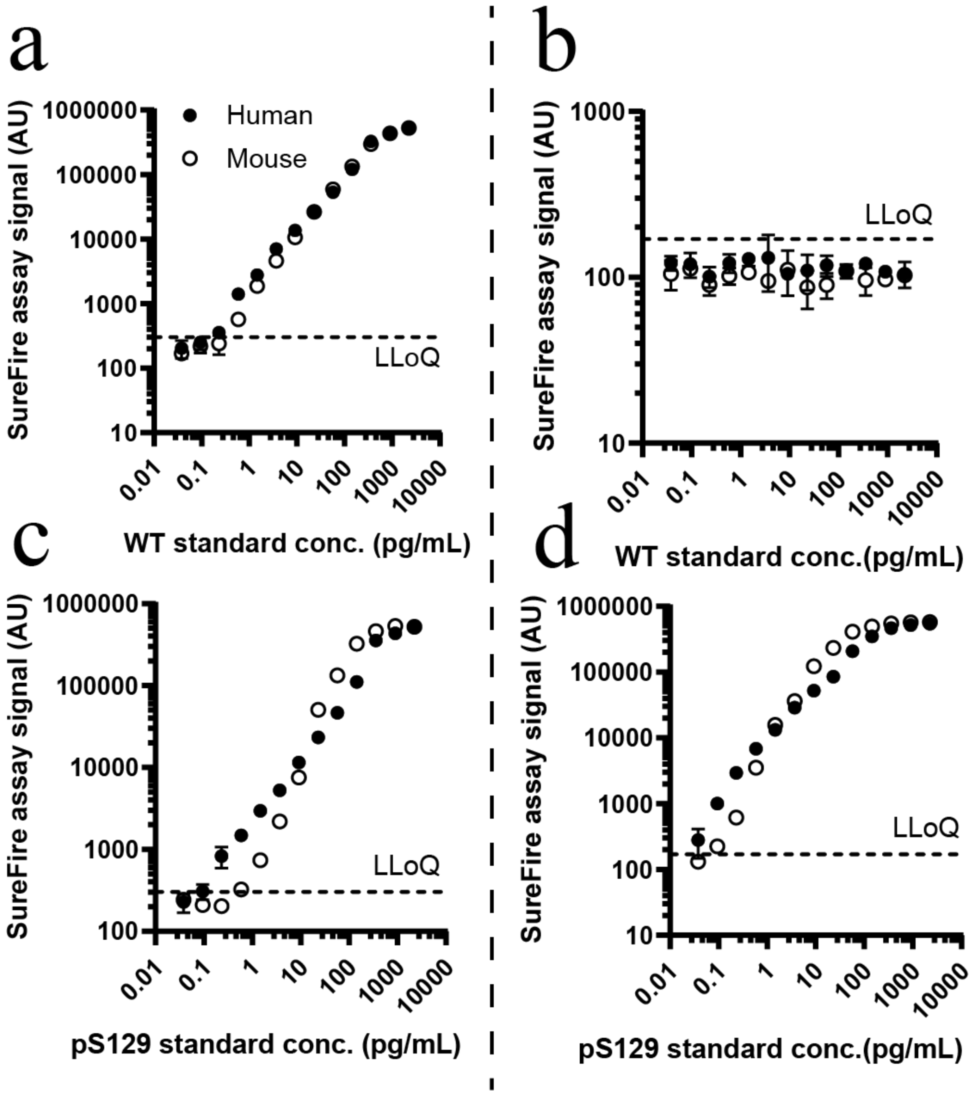

Supplementary Figure 2. Characterization of the linear dynamic range of new SureFire Ultra assays using purified protein standards. The linear dynamic range of the most sensitive new formulations of the new total (left; #ALSU-TASYN-B, Revvity) and pS129 (right; #ALSU-PASYN-B, Revvity) α-synuclein assay were characterized using purified human (Hu) and mouse (Ms) wild-type (WT; **a-b**) and pS129 (**c-d**) α-synuclein standards. All associated data are contained within Supplementary Tables 2-3. The lower limit of quantification (LLoQ) was defined as 6 standard deviations above the mean of the blank. Data represent mean ± standard deviation. AU, arbitrary units.

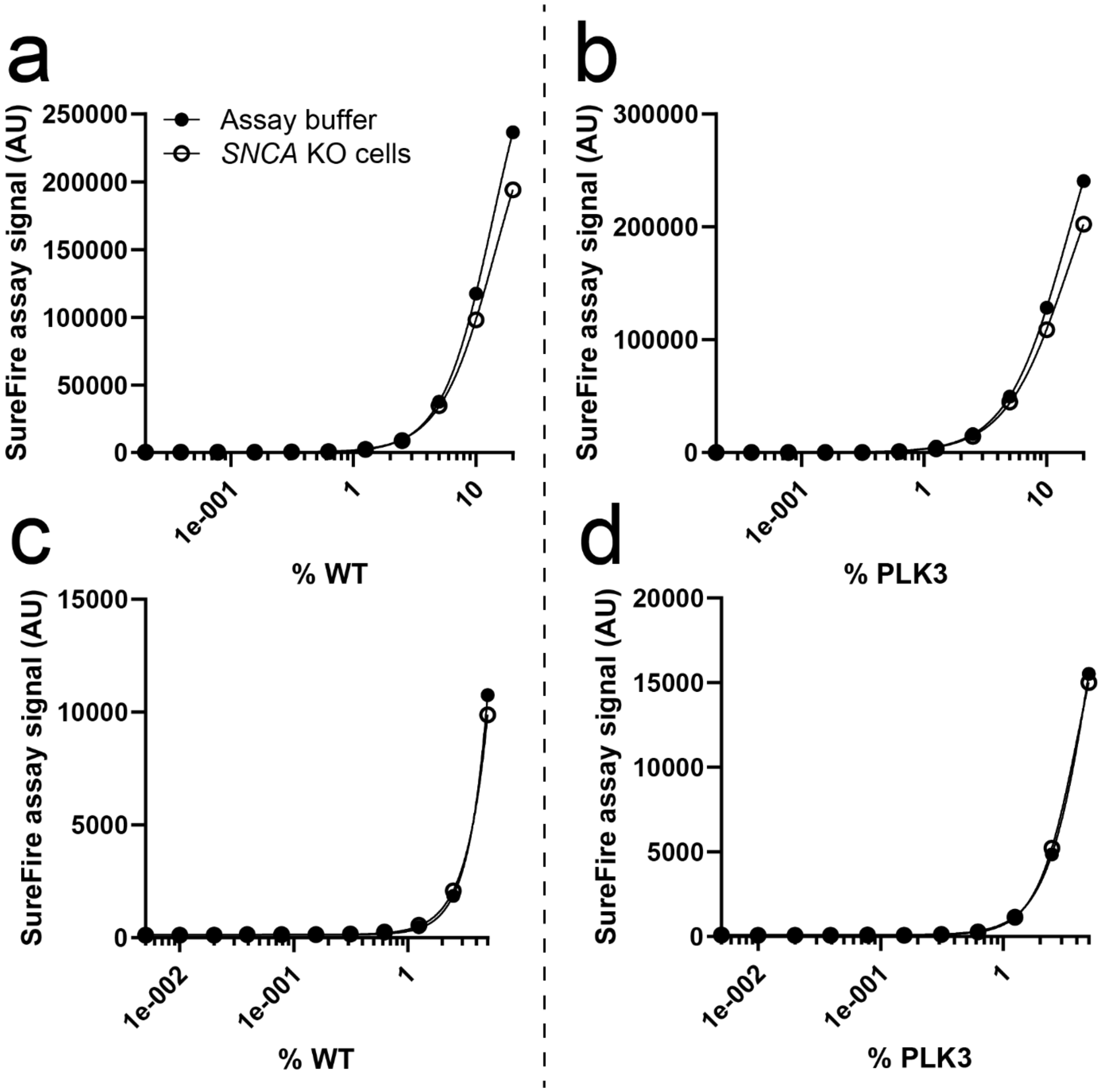

Supplementary Figure 3. Matrix effects in HEK293 cell lysate using a parallelism experiment. Matrix effects were measured for the most sensitive new total (left; #ALSU-TASYN-B, Revvity) and pS129 (right; #ALSU-PASYN-B, Revvity) α-synuclein assay formulation using 5-fold (**a, b**) and 20-fold (**c, d**) diluted wild-type (WT) and polo-like kinase 3 (PLK3)-transfected HEK293 cell lysates, respectively, diluted in either *SNCA* knock-out (KO) HEK293 cell lysate of an equivalent dilution (5- or 20-fold) or assay buffer. AU, arbitrary units.

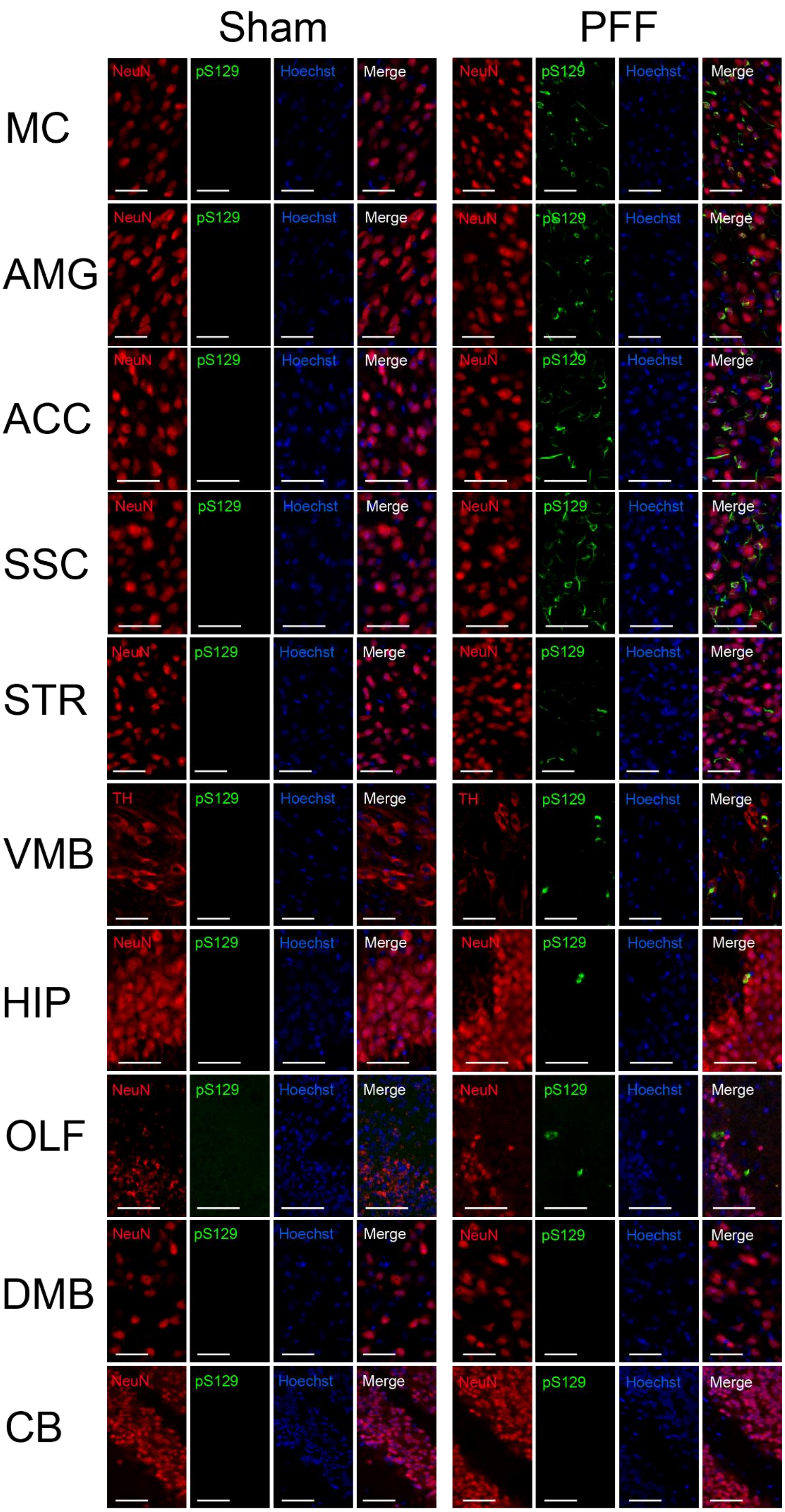

Sham
PFF
MC
AMG
ACC
SSC
STR
VMB
HIP
OLF
DMB
CB
NeuN
pS129
Hoechst
Merge
TH

Supplementary Figure 4. Immunohistochemical profiles of pS129 α-synuclein across brain regions of interest in sham and PFF mice. Immunofluorescent labelling of pS129 α-synuclein (EP1536Y, Abcam) was performed in 30μm free-floating, fixed coronal brain tissue sections from 3-month-old PFF (*n* = 3) and sham mice (*n* = 3). Neurons were labelled with neuronal nuclear protein (NeuN; ab279297, Abcam) or tyrosine hydroxylase (TH; ab76442, Abcam) and Hoechst used to counterstain all cell nuclei. Scale bars represent 50μm. ACC, anterior cingulate cortex; AMG, amygdala; CB, cerebellum; DMB, dorso-medial midbrain; HIP, hippocampus; MC, motor cortex; OLF, olfactory bulb; SSC, somatosensory cortex; STR, striatum; VMB, ventral midbrain.

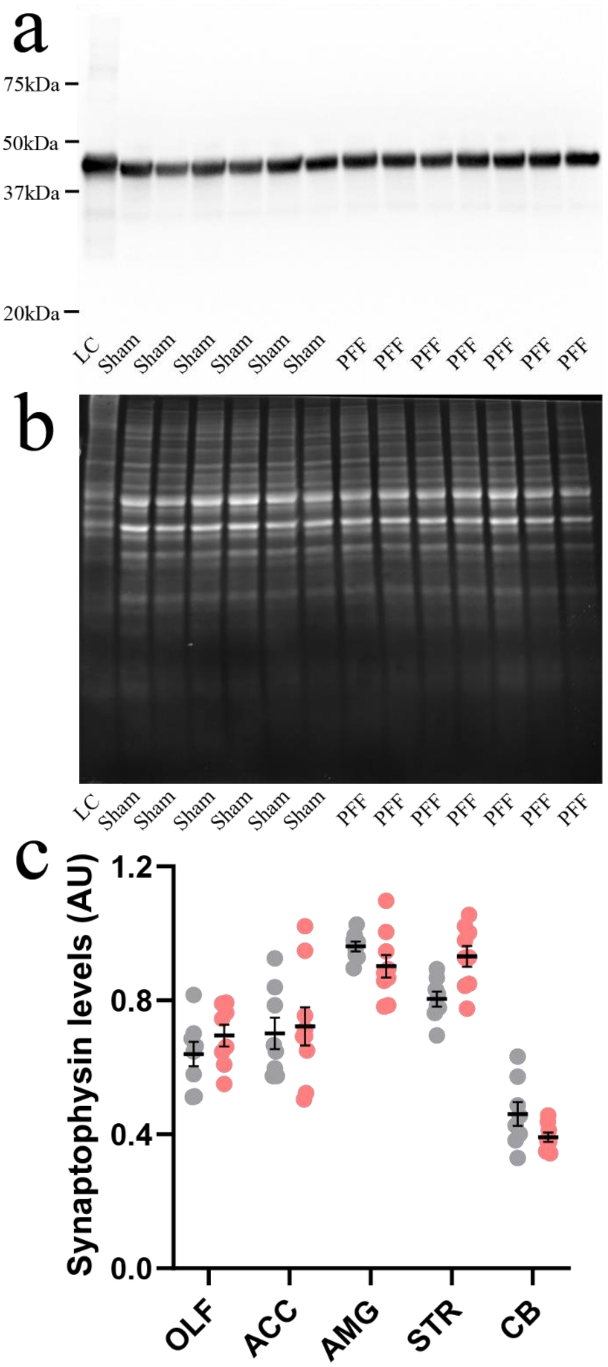

Supplementary Figure 5. Immunoblot quantification of synaptophysin in sham and PFF mouse brain regions. Synaptophysin immunoblotting (YE269, Abcam) identified a single synaptophysin isoform (**a**), which was quantified using densitometry and normalized to Sypro Ruby total protein levels to correct against any differences in protein loading between samples (**b**). Representative full blot images above display data from the striatum. Levels of synaptophysin protein were quantified in immunoblots of whole tissue extracts from the olfactory bulb (OLF), anterior cingulate cortex (ACC), amygdala (AMG), striatum (STR) and cerebellum (CB) of sham (n = 8) and PFF (n = 9) mice (**c**). Data represent mean ± standard deviation. AU, arbitrary units. No significant differences were identified between corresponding regions of the sham and PFF mouse brain using a Two-way ANOVA paired with Sidak's multiple comparisons post-hoc tests.

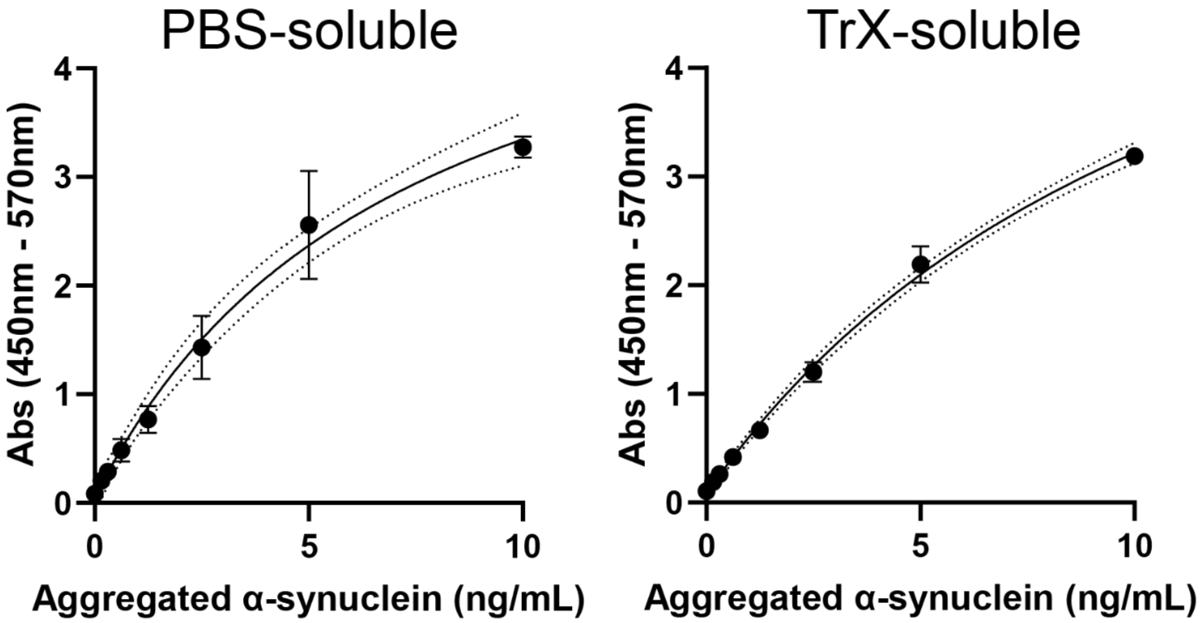

Supplementary Figure 6. Characterization of the sensitivity of the Biolegend LEGEND MAX α-Synuclein Aggregate ELISA using a purified α-synuclein aggregate standard. Curves were generated using the aggregated α-synuclein standard provided with the assay following its serial dilution in either phosphate buffered saline (PBS) or Triton X-100 (Triton) fraction buffers. Final assay signal was calculated by subtracting the absorbance (Abs) signal at 570nm from that obtained at 450nm. Datapoints represent mean ± SEM for each concentration (n = 3) with dotted lines depicting the 95% confidence interval for that value.

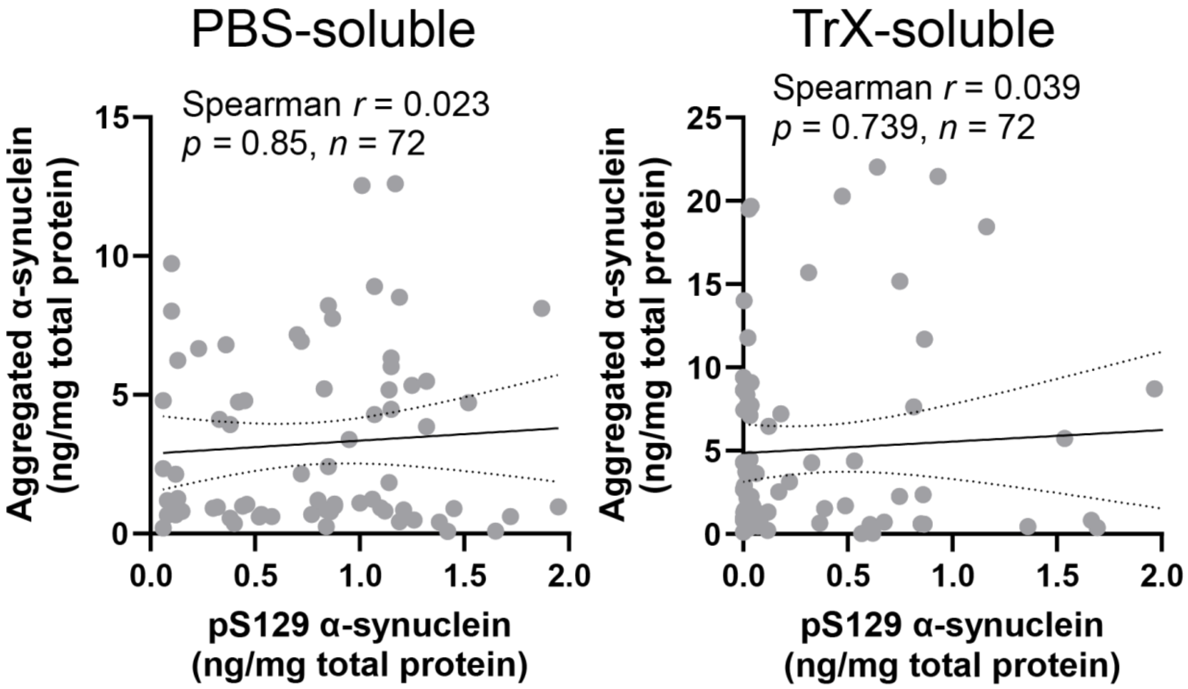

Supplementary Figure 7. Correlations between levels of aggregated and pS129 α-synuclein in PBS- and TrX-soluble fractions. Correlations only include data from brain regions of moderate (*n* = 4) or severe (*n* = 4) pS129 α-synuclein pathology in PFF mice (*n* = 9). Details of statistical comparisons are presented within panels.

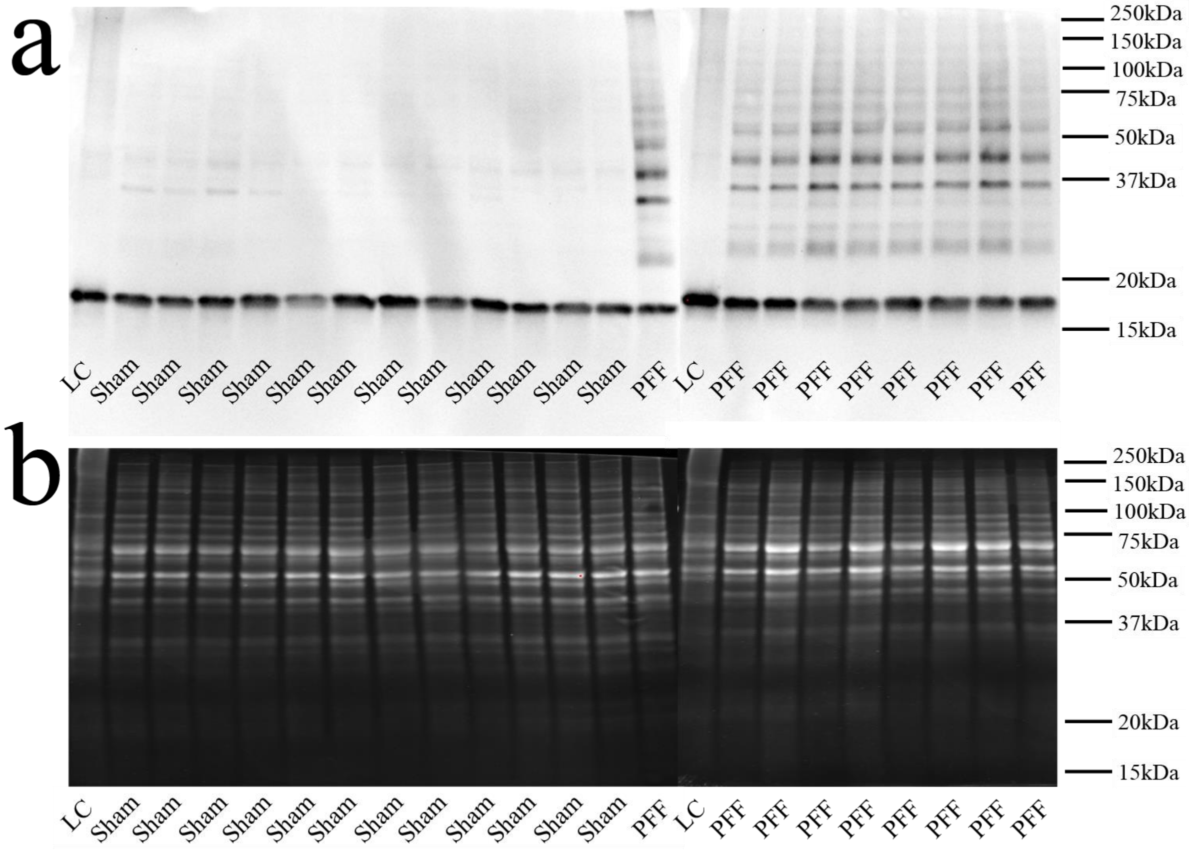

Supplementary Figure 8. Full representative immunoblots characterising α-synuclein phosphorylation in brain tissue extracts from sham and PFF mice. α-Synuclein S129 phosphorylation in the anterior cingulate cortex (ACC), striatum and cerebellum of sham and PFF mice was examined using immunoblotting (**a**; D1R1R antibody, Cell Signalling). Immunoblots above are from the ACC only. pS129 α-synuclein band intensities were normalized to Sypro Ruby total protein staining to correct against any differences in protein loading between samples (**b**). A loading control (LC) consisting of pooled sham mouse brain tissue extracts was included on each gel to correct against any differences between immunoblot batches.

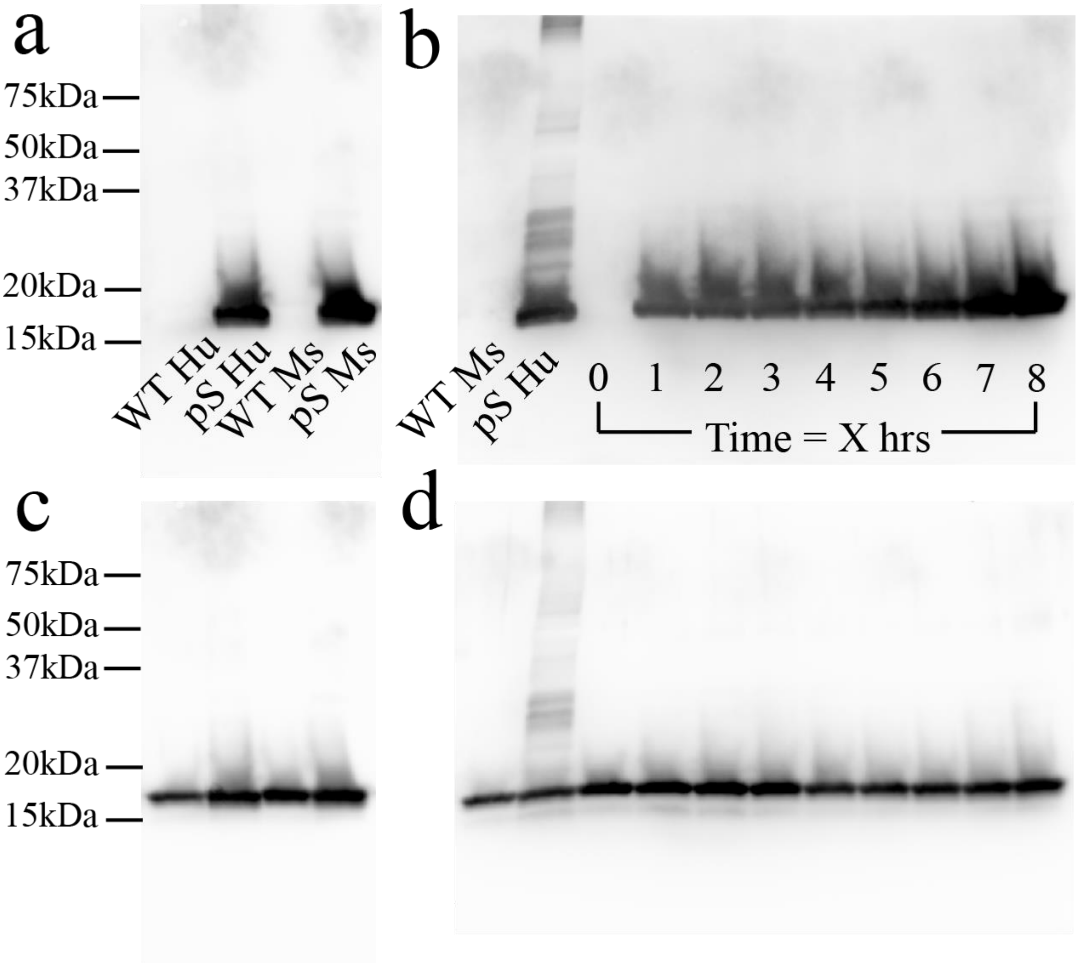

Supplementary Figure 9. Full representative immunoblots characterising S129 phosphorylation in purified protein standards. The S129 phosphorylation status of these samples was determined using immunoblotting against human (Hu) and mouse (Ms) pS129 (pS) α-synuclein (D1R1R, Cell Signalling) (**a**). This approach was also used to establish successful phosphorylation of mouse α-synuclein S129 over an 8-hour incubation with human polo-like kinase 3 (PLK3) in kinase reaction buffer (details outlined in methods) (**b**). In both experiments, membranes were stripped and re-probed for human and mouse wild-type (WT) α-synuclein (Syn1, BD Biosciences) to confirm the presence of non-phosphorylated α-synuclein in control lanes (**c, d**), and to demonstrate equal gel loading of α-synuclein over the PLK3 time course experiment (**d**).

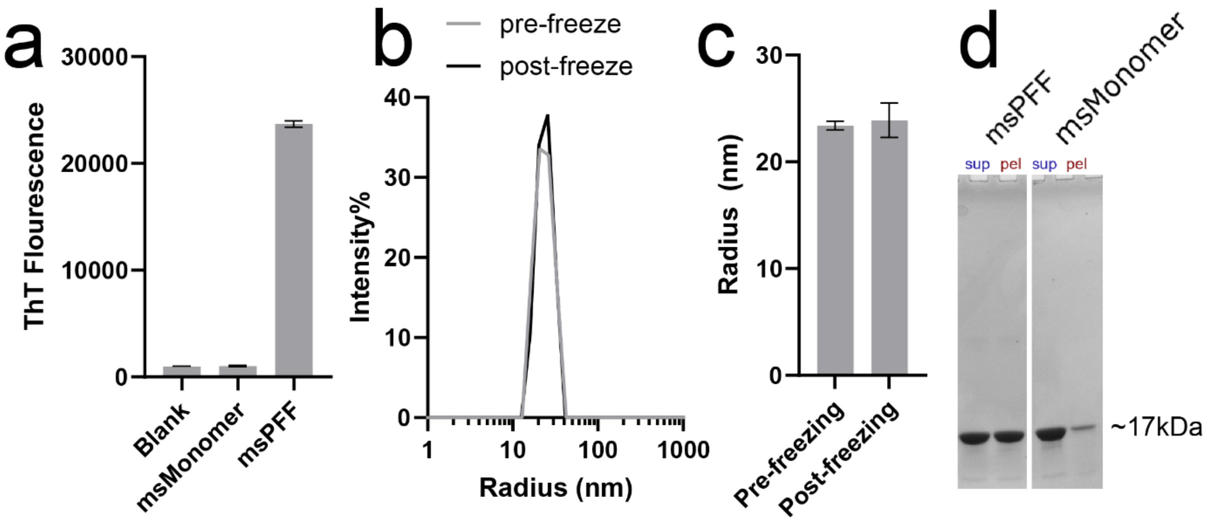

Supplementary Figure 10. Characterisation of mouse pre-formed fibril (PFF) structure and solubility. The amyloid structure of mouse α-synuclein PFFs (msPFF) compared with native monomeric mouse α-synuclein (msMonomer) was confirmed using a Thioflavin-T binding assay (**a**). Their fragment size did not change upon freeze-thaw using dynamic light scattering measurements (DynaPro NanoStar, Wyatt Technology) (**b, c**). Sedimentation analysis revealed that approximately 50% of the PFFs became soluble after sonication and freeze/thaw (**d**).

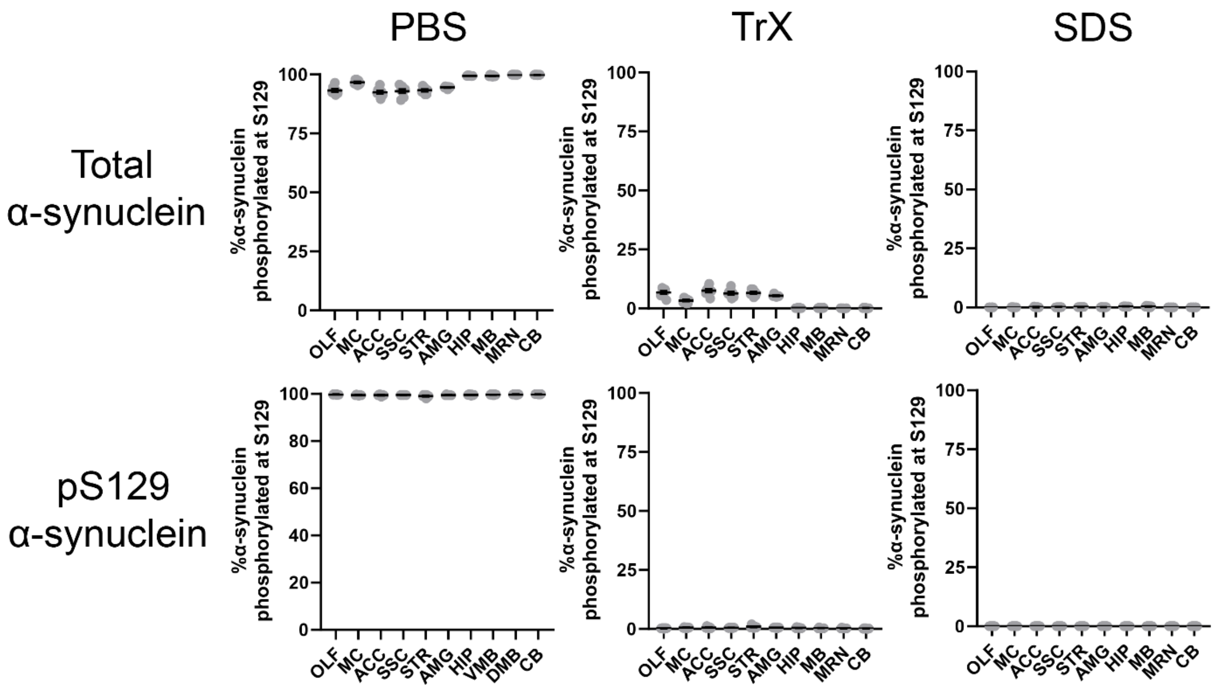

Supplementary Figure 11. Proportions of total and pS129 α-synuclein in PBS, TrX and SDS fractions of sham mouse brain. Datapoints represent the mean analyte quantity for a given sample, which was calculated from triplicate measurements of that sample in the same assay plate. Error bars represent mean ± standard error of the mean for that sample group.

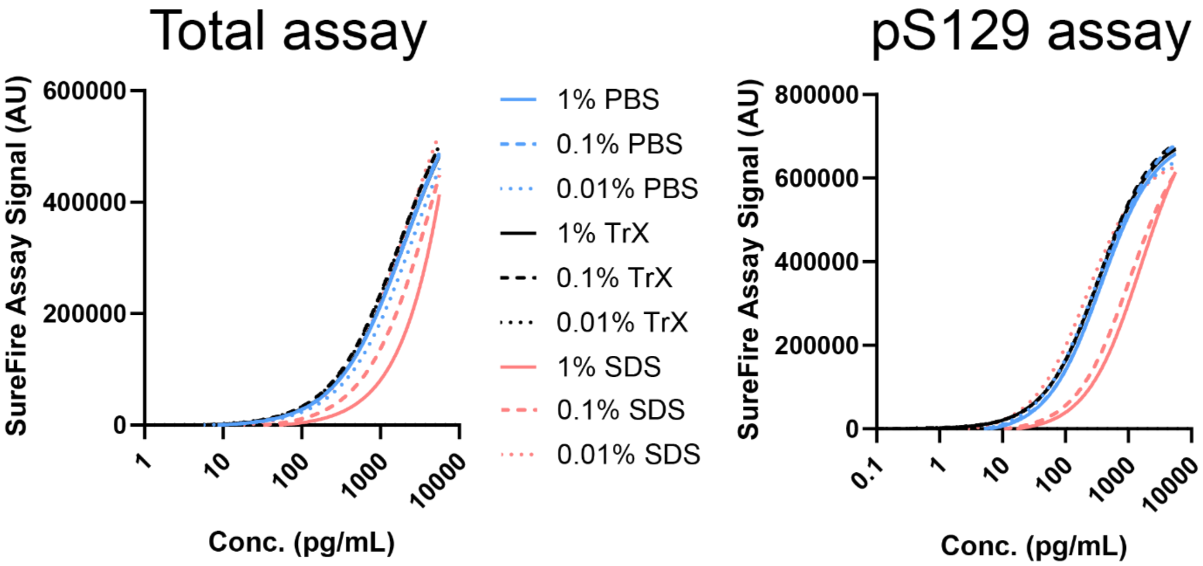

Supplementary Figure 12. Evaluation of the impact of PBS, TrX and SDS on assay performance. For this experiment, 1%, 0.1% and 0.01% solutions of PBS, TrX and SDS were generated in 1x assay lysis buffer, and then used to serially dilute WT and pS129 α-synuclein standards, which were measured using the total and pS129 assay respectively. Any buffer interferences are reflected as rightwards shifts in the dilution curves of these standards. PBS and TrX did not impact the performance of the total or pS129 assay at any concentration tested, while SDS significantly dampened assay signal at concentrations between 1-to-0.1%. Data represent line of best fit, which was generated from mean and standard deviation measurements using non-linear regression.

Supplementary Table 1. Characterization of previous total and pS129 α-synuclein SureFire Ultra assay formulations using purified protein standards.

Sample	Protein conc. (pg/mL)	ALSU-TASYN-A total α-synuclein assay (LOD: 156.7, LOQ: 174.4)			ALSU-PASYN-A pS129 α-synuclein assay (LOD: 145.7, LOQ: 173.9)		
		Mean	SD	Inter-assay RSD	Mean	SD	Inter-assay RSD
Blank	0	139.0	5.9	4.2	117.5	9.4	8.0
Mouse pS129 α-synuclein	0.094	-	-	-	135.0	9.5	7.0
	0.23	135.0	10.1	7.5	123.0	7.2	5.9
	0.59	145.0	12.2	8.4	144.0	4.5	3.1
	1.47	139.0	21.5	15.5	158.0	10.5	6.6
	3.67	135.0	9.8	7.3	359.0	33.1	9.2
	9.18	137.0	20.0	14.6	594.0	40.5	6.8
	22.94	148.0	23.7	16.0	1862.0	23.0	1.2
	57.34	149.0	11.1	7.4	7441.0	124.8	1.7
	143.36	136.0	35.2	25.9	30567.0	1255.2	4.1
	358.4	139.0	16.3	11.7	75618.0	998.4	1.3
	896	142.0	10.6	7.5	175823.0	11153.1	6.3
	2240	147.0	10.6	7.2	310631.0	5396.7	1.7
	5600	156.0	5.2	3.3	356138.0	16296.8	4.6
	14000	153.0	4.6	3.0	-	-	-
	28000	167.0	19.0	11.4	-	-	-
	56000	175.0	3.8	2.2	-	-	-
Mouse WT α-synuclein	0.094	-	-	-	119	7.5	6.3
	0.23	156	4.4	2.8	135	8.3	6.1
	0.59	167	2.3	1.4	137	12.8	9.3
	1.47	172	6.7	3.9	148	26.1	17.6
	3.67	179	6.1	3.4	129	11.2	8.7
	9.18	172	2.9	1.7	136	4.5	3.3
	22.94	172	6.7	3.9	139	4.6	3.3
	57.34	175	8.9	5.1	122	11	9.0
	143.36	161	4	2.5	147	9.8	6.7
	358.4	176	6.7	3.8	116	13.2	11.4
	896	210	26.7	12.7	133	41.2	31.0
	2240	280	4	1.4	137.0	25.6	18.7
	5600	293.0	2.1	0.7	175.0	43.7	25.0
	14000	647.0	67.0	10.4	-	-	-
	28000	1414.0	98.4	7.0	-	-	-
	56000	3263.0	91.7	2.8	-	-	-
Human pS129 α-synuclein	0.094	-	-	-	149	9.9	6.6
	0.23	119.0	4.0	3.4	110	12.1	11.0
	0.59	132.0	5.2	3.9	123	10.5	8.5
	1.47	187.0	6.9	3.7	125	8.5	6.8
	3.67	242.0	8.9	3.7	115	12.5	10.9
	9.18	734.0	16.0	2.2	105	11.2	10.7
	22.94	2209.0	61.7	2.8	129	13.2	10.2
	57.34	3866.0	150.7	3.9	123	11.2	9.1
	143.36	9648.0	820.8	8.5	126	0.6	0.5
	358.4	21824.0	1569.6	7.2	99	8.7	8.8
	896	85624.0	5052.7	5.9	110	4.6	4.2
	2240	187110.0	10976.3	5.9	102.0	10.5	10.3
	5600	318475.0	25955.1	8.1	511.0	36.2	7.1
	14000	360226.0	29371.4	8.2	-	-	-
	28000	368963.0	15569.5	4.2	-	-	-
	56000	370536.0	17555.3	4.7	-	-	-

Human WT α-synuclein	0.094	-	-	-	112	3.6	3.2
	0.23	124.0	3.0	2.4	121	2.3	1.9
	0.59	134.0	4.5	3.4	147	5.2	3.5
	1.47	144.0	7.1	4.9	172	11.1	6.5
	3.67	173.0	14.0	8.1	113	20.9	18.5
	9.18	224.0	10.0	4.5	116	12.1	10.4
	22.94	321.0	33.3	10.4	120	19.3	16.1
	57.34	640.0	52.8	8.3	145	10.6	7.3
	143.36	1278.0	312.6	24.5	120	10.6	8.8
	358.4	2308.0	420.8	18.2	123	17.2	14.0
	896	5836.0	1110.5	19.0	123	8.9	7.2
	2240	22559.0	2050.0	9.1	120.0	8.9	7.4
	5600	85978.0	8426.3	9.8	145.0	9.8	6.8
	14000	234311.0	15926.1	6.8	-	-	-
	28000	336622.0	26371.3	7.8	-	-	-
	56000	359868.0	19563.1	5.4	-	-	-

Existing formulations of the total (#ALSU-TASYN-A, Revvity) and pS129 (#ALSU-PASYN-A, Revvity) α-synuclein assay were characterized using purified human and mouse wild-type (WT) and pS129 α-synuclein standards. Inter-assay variation (RSD) was defined as the standard deviation expressed as a percentage of the mean. The limit of detection (LoD) and lower limit of quantification (LLoQ) were defined as 3 and 6 standard deviations above the mean of the blank, respectively. Data represent raw assay signal measurements in arbitrary units.

Supplementary Table 2. Characterization of new total and pS129 α-synuclein SureFire Ultra assay formulations using purified protein standards.

α-syn std	Standard conc. (ng/mL)	New total α-synuclein assay formulations					New pS129 α-synuclein assay formulations				
		1	2	3	4	5	1	2	3	4	5
Mouse p-S129	20	840792	1029656	742576	341	1593	998587	756485	1067388	938	486616
		910771	954597	693577	290	1391	939358	734843	964689	975	479148
	2	277935	194720	129172	197	743	327756	193746	481042	415	75927
		285257	194973	129277	231	559	326086	194712	483272	378	73662
	0.2	6079	1891	4855	205	244	22037	17900	29594	222	4674
		7006	1326	4759	172	243	20228	16816	28332	214	4401
	0	303	273	306	210	180	281	433	324	210	243
		323	256	276	193	235	176	240	219	193	184
Mouse WT	20	657404	742985	480785	201	205	218	290	252	151	201
		644834	739840	482842	256	168	226	260	210	201	252
	2	94995	25682	42098	240	159	235	218	189	197	201
		95535	21375	38472	264	189	189	243	189	168	180
	0.2	2303	595	2286	197	168	213	264	193	180	243
		1770	518	2343	227	202	201	239	227	184	168
	0	214	227	277	210	176	210	268	210	168	184
		218	243	239	277	197	193	222	210	205	243
Human p-S129	20	1340	811240	665	175812	849430	1165	576515	881121	692920	465942
		1242	824176	590	190495	872589	1158	595343	871329	675019	462341
	2	317	67561	271	2762	134524	405	112253	347305	175893	72925
		251	62621	267	2184	136104	326	103457	301306	164545	67799
	0.2	196	1168	268	254	3117	246	12435	27363	19077	8347
		188	1093	225	288	2676	225	13363	30153	22601	9179
	0	213	225	230	213	196	196	263	267	259	221
		376	228	284	841	200	221	238	180	188	205
Human WT	20	1474	663827	523	134131	771906	301	247	230	230	221
		1485	670452	552	132822	722961	334	263	188	225	196
	2	306	22796	280	1676	57367	234	221	213	184	217
		268	20476	259	1337	52731	209	213	209	200	234
	0.2	222	427	242	255	788	192	234	188	188	175
		188	373	272	188	683	209	200	201	171	180
	0	217	276	268	205	213	196	406	205	184	225

		213	264	242	238	201	222	234	205	201	196

Five new formulations of the total and pS129 α-synuclein assay were characterized using purified human and mouse wild-type (WT) and pS129 α-synuclein standards. Data represent mean raw assay signal measurements in arbitrary units.

Supplementary Table 3. Characterization of the most sensitive new total and pS129 α-synuclein SureFire Ultra assay formulations using purified protein standards.

Sample	Protein conc. (pg/mL)	New Total α-synuclein pairing 2 (LOD: 233.1, LOQ: 304.2)			New pS129 α-synuclein pairing 3 (LOD: 150.7, LOQ: 192.1)		
		Mean	SD	Inter-assay RSD	Mean	SD	Inter-assay RSD
Blank	0	162.0	23.7	14.6	109.3	13.8	12.6
Mouse pS129 α-synuclein	0.038	200.0	13.3	6.7	132.0	2.0	1.5
	0.094	203.0	25.1	12.4	224.0	7.8	3.5
	0.23	325.0	24.7	7.6	617.0	58.7	9.5
	0.59	640.0	30.4	4.8	3523.0	97.5	2.8
	1.47	2190.0	57.9	2.6	15815.0	176.1	1.1
	3.67	7583.0	363.9	4.8	36411.0	919.1	2.5
	9.18	50570.0	3329.9	6.6	122497.0	1745.1	1.4
	22.94	133627.0	12612.2	9.4	234623.0	3148.9	1.3
	57.34	323984.0	10499.0	3.2	412329.0	13342.7	3.2
	143.36	462248.0	7490.4	1.6	495793.0	3648.9	0.7
	358.4	533905.0	16465.8	3.1	548751.0	38523.1	7.0
	896	524503.0	37267.9	7.1	570531.0	22952.3	4.0
	2240	543322.0	47032.3	8.7	574021.0	26438.8	4.6
Mouse WT α-synuclein	0.038	169	13.7	8.1	105	21.5	20.5
	0.094	219	46.4	21.2	113	5	4.4
	0.23	241	79	32.8	90	12.1	13.4
	0.59	568	9.3	1.6	102	11.6	11.4
	1.47	1869	123.5	6.6	107	9.3	8.7
	3.67	4619	405.5	8.8	95	8.1	8.5
	9.18	10629	926.1	8.7	111	33.6	30.3
	22.94	26534	1910.8	7.2	87	22.6	26.0
	57.34	59111	4623.2	7.8	90	15.6	17.3
	143.36	134113	11574.5	8.6	109	9.3	8.5
	358.4	298003	17099.7	5.7	96	18.2	19.0
	896	435612	23564	5.4	97	5.2	5.4
	2240	526356	35263	6.7	103	4.5	4.4
Human pS129 α-synuclein	0.038	228	59.4	26.1	280	132.5	47.3
	0.094	308	65.6	21.3	1004	28.3	2.8
	0.23	831	239.4	28.8	2935	108.5	3.7
	0.59	1489	120.5	8.1	6834	367.8	5.4
	1.47	2960	486.1	16.4	13260	1939.7	14.6
	3.67	5270	518.5	9.8	28740	2849.7	9.9
	9.18	11565	461	4.0	52311	7528.7	14.4
	22.94	23302	2141	9.2	85369	2123.3	2.5
	57.34	46691	1640.3	3.5	208784	30487	14.6
	143.36	111313	11016	9.9	350602	12652.2	3.6
	358.4	356486	19709.8	5.5	468104	4513	1.0
	896	434334	56644	13.0	516624	28545.8	5.5
	2240	518737	32622.4	6.3	545949	13291.6	2.4
Human WT α-synuclein	0.038	205	63.3	30.9	122	12.1	9.9
	0.094	253	59	23.3	120	20.6	17.2
	0.23	357	12.1	3.4	101	14.4	14.3
	0.59	1403	212.3	15.1	122	15.6	12.8
	1.47	2782	235.7	8.5	129	12.1	9.4
	3.67	7048	684.5	9.7	131	49	37.4
	9.18	13682	1689.8	12.4	105	7.1	6.8
	22.94	25904	2414.4	9.3	110	26.7	24.3
	57.34	53230	5042.2	9.5	118	16.9	14.3

	143.36	120243	15425.4	12.8	109	10.4	9.5
	358.4	326893	15281.9	4.7	121	4.5	3.7
	896	449586	23526	5.2	108	4.5	4.2
	2240	522986	29653.2	5.7	105	18.7	17.8

The most sensitive new total (#ALSU-TASYN-B, Revvity) and pS129 (#ALSU-PASYN-B, Revvity) α-synuclein assay formulation were characterized using purified human and mouse wild-type (WT) and pS129 α-synuclein standards. Inter-assay variation (RSD) was defined as the standard deviation expressed as a percentage of the mean. The limit of detection (LoD) and lower limit of quantification (LLoQ) were defined as 3 and 6 standard deviations above the mean of the blank, respectively. Data represent raw assay signal measurements in arbitrary units.

Supplementary Table 4. Characterization of new total and pS129 α-synuclein SureFire Ultra assays using extracts from mouse brain tissues and HEK293 cells.

Sample	Dilution factor	New Total α-synuclein pairing 2 (LOD: 233.1, LOQ: 304.2)			New total α-synuclein pairing 3 (LOD: 259.4, LOQ: 391.1)			New pS129 α-synuclein pairing 3 (LOD: 150.7, LOQ: 192.1)			New pS129 α-synuclein pairing 2 (LOD: 141.2, LOQ: 179.5)		
		Mean	SD	Inter-assay RSD	Mean	SD	Inter-assay RSD	Mean	SD	Inter-assay RSD	Mean	SD	Inter-assay RSD
WT mouse brain tissue	Neat	519383.0	20653.2	4.0	477170.0	18897.9	4.0	128664.0	1074.7	0.8	31413.0	2453.8	7.8
	2	527502.0	6619.0	1.3	491094.0	4284.9	0.9	126258.0	8867.9	7.0	31082.0	5546.3	17.8
	4	529393.0	29850.5	5.6	486487.0	23452.7	4.8	124355.0	10461.7	8.4	31520.0	5469.4	17.4
	8	525027.0	49416.3	9.4	484084.0	8826.4	1.8	119105.0	3926.0	3.3	28206.0	2488.5	8.8
	16	528122.0	13042.0	2.5	477008.0	11361.4	2.4	103232.0	6748.2	6.5	29853.0	2577.5	8.6
	32	515598.0	7265.3	1.4	486933.0	35996.6	7.4	72292.0	1043.0	1.4	30285.0	1248.8	4.1
	64	518405.0	12465.6	2.4	453018.0	21384.7	4.7	37331.0	878.8	2.4	19466.0	1837.6	9.4
	128	504747.0	14532.4	2.9	409767.0	7796.9	1.9	15619.0	616.8	3.9	11628.0	569.8	4.9
	256	485607.0	15069.2	3.1	338408.0	16359.1	4.8	5091.0	88.6	1.7	6000.0	459.1	7.7
	512	422613.0	6398.5	1.5	241884.0	324.8	0.1	1561.0	90.6	5.8	3119.0	158.6	5.1
	1024	284070.0	17109.5	6.0	146147.0	6118.5	4.2	538.0	42.4	7.9	1552.0	65.5	4.2
	2048	132522.0	6133.0	4.6	70906.0	1376.3	1.9	237.0	14.3	6.0	806.0	66.9	8.3
	4096	49527.0	1159.6	2.3	36135.0	749.9	2.1	148.0	5.8	3.9	515.0	19.0	3.7
	8192	15882.0	1754.0	11.0	15976.0	439.9	2.8	107.0	2.9	2.7	356.0	13.7	3.8
	16384	3286.0	96.4	2.9	5876.0	211.6	3.6	101.0	11.1	11.0	248.0	29.5	11.9
	32768	725.0	56.1	7.7	1919.0	40.0	2.1	91.0	16.2	17.8	177.0	31.8	18.0
	Blank	126	9.0	7.1	142	24.9	17.5	90	12.1	13.5	105	10.4	9.9
Snca-/- mouse brain tissue	Neat	1789	147.2	8.2	1940	239.1	12.3	89	4.5	5.1	97	5.2	5.4
	2	1332	60.4	4.5	1209	107.5	8.9	100	25.9	25.9	88	5.2	5.9
	4	755	120	15.9	1143	100.6	8.8	70	13.7	19.6	95	10.4	10.9
	8	406	67	16.5	920	142.5	15.5	76	13.5	17.8	112	15.9	14.2
	16	363	32.3	8.9	805	160.5	19.9	103	4.5	4.4	107	19.2	17.9
	32	238	26.1	11.0	677	130.5	19.3	116	47.1	40.6	119	13.7	11.5
	64	189	22.6	12.0	554	73.6	13.3	108	22.1	20.5	114	25.2	22.1
	128	146	13.7	9.4	355	67.5	19.0	110	13	11.8	120	15.6	13.0
	256	122	14.4	11.8	247	31.6	12.8	113	27	23.9	129	18	14.0
	512	120	22	18.3	201	7.8	3.9	92	5.2	5.7	99	11.1	11.2
	1024	98	9	9.2	153	27.5	18.0	113	6.7	5.9	99	13.7	13.8

	2048	111	18	16.2	144	8.9	6.2	96	18.2	19.0	122	12.7	10.4
	4096	126	8.9	7.1	140	11.6	8.3	96	20.4	21.3	113	10.7	9.5
	Blank	121	10.1	8.3	126	18.7	14.9	93	9.0	9.7	108	6.6	6.1
WT HEK293 cells	Neat	458592	16881.9	3.7	4531	488.6	10.8	10856	504.9	4.7	7946	149	1.9
	2	467632	3642.1	0.8	2243	501	22.3	6407	958.2	15.0	6821	222.7	3.3
	4	440610	15093.4	3.4	720	62.4	8.7	2621	68.5	2.6	3863	211.2	5.5
	8	371134	12992.4	3.5	304	23.1	7.6	865	146.3	16.9	2029	76.6	3.8
	16	266379	10172.9	3.8	222	16.5	7.4	313	18.8	6.0	1220	23.3	1.9
	32	130916	6764.5	5.2	155	18.2	11.7	172	36.9	21.5	690	17	2.5
	64	46238	3589.1	7.8	162	4.5	2.8	128	13.3	10.4	411	38.2	9.3
	128	9315	1828.2	19.6	170	2.6	1.5	142	21	14.8	281	16.5	5.9
	256	2184	184.1	8.4	165	14.3	8.7	127	21.5	16.9	236	9.5	4.0
	512	607	41.7	6.9	155	17.2	11.1	117	13.5	11.5	162	7.5	4.6
	1024	313	73.7	23.5	174	9.3	5.3	105	11.6	11.0	144	20.8	14.4
	2048	230	31.2	13.6	189	25.3	13.4	130	27.2	20.9	141	6.7	4.8
	4096	203	11.7	5.8	180	29.7	16.5	106	13.7	12.9	132	14.4	10.9
	Blank	169	12.7	7.5	133	9.5	7.1	91	14.6	16.0	127	10.4	8.2
PLK3-transfected HEK293 cells	Neat	342392	9226.1	2.7	2531	350	13.8	150754	6274.2	4.2	120563	7985.1	6.6
	2	327276	2669.2	0.8	2051	600	29.3	127962	2422	1.9	100548	3564.5	3.5
	4	276227	2739.1	1.0	532	55	10.3	66400	1044.1	1.6	56213	2563.2	4.6
	8	225865	1241.1	0.5	295	15	5.1	26010	1297.2	5.0	20356	1985.6	9.8
	16	148819	4469.7	3.0	275	26	9.5	8887	392.6	4.4	6594	562.4	8.5
	32	62637	6088.7	9.7	154	28	18.2	2413	169.8	7.0	2256	156.9	7.0
	64	18720	484	2.6	165	5.1	3.1	756	42.2	5.6	859	56.9	6.6
	128	4274	303.7	7.1	162	3.2	2.0	261	13.3	5.1	302	5.2	1.7
	256	991	84.6	8.5	166	5.3	3.2	130	10.4	8.0	152	15.2	10.0
	512	317	11.8	3.7	156	6.2	4.0	114	7.1	6.2	140	11.2	8.0
	1024	166	14.7	8.9	153	19.2	12.5	115	24.9	21.7	141	25	17.7
	2048	127	10.8	8.5	165	2.1	1.3	90	11.6	12.9	135	16.2	12.0
	4096	123	16.3	13.3	168	7.5	4.5	95	9.8	10.3	140	12.3	8.8
	Blank	122	19.1	15.6	125	21.0	16.8	73	9.0	12.3	128	12.1	9.4
SNCA-/- HEK293 cells	Neat	427	47.5	11.1	494	72	14.6	120	29.5	24.6	461	25.7	5.6
	2	320	22.4	7.0	377	63.7	16.9	120	13.7	11.4	359	25.2	7.0
	4	252	57	22.6	182	15.5	8.5	93	2.9	3.1	279	14.9	5.3
	8	232	23.1	10.0	161	12.3	7.6	86	13.5	15.7	179	14.4	8.0
	16	140	11.6	8.3	136	9.5	7.0	84	6.7	8.0	171	8.1	4.7

	32	127	6.7	5.3	121	19.5	16.1	83	10.4	12.5	125	2.3	1.8
	64	111	12.1	10.9	167	16.4	9.8	90	11.7	13.0	117	15.6	13.3
	128	176	16.1	9.1	156	2.9	1.9	92	18.2	19.8	117	14	12.0
	256	152	21.5	14.1	153	17.1	11.2	90	20.1	22.3	115	7.6	6.6
	512	128	6.7	5.2	141	17.4	12.3	90	11.7	13.0	120	27.1	22.6
	1024	126	20.3	16.1	167	31.6	18.9	105	22.1	21.0	109	5.2	4.8
	2048	165	6.7	4.1	132	13	9.8	91	16	17.6	105	6.7	6.4
	4096	121	15.6	12.9	105	2.9	2.8	81	4.5	5.6	125	27.5	22.0
	Blank	126	16.5	13.1	123	6.7	5.4	96	22.0	23	118	22.6	19.2

The 2 most sensitive new total and pS129 α-synuclein assay formulations were characterized using extracts from wild-type (WT) mouse brain tissue and HEK293 cells, *Snca* knock-out mouse brain tissues, polo-like kinase 3 (PLK3)-transfected HEK293 cells, and *SNCA* knock-out HEK293 cells. Extracts were serially-diluted using assay buffer. Inter-assay variation (RSD) was defined as the standard deviation expressed as a percentage of the mean. The limit of detection (LoD) and lower limit of quantification (LLoQ) were defined as 3 and 6 standard deviations above the mean of the blank, respectively. Data represent raw assay signal measurements in arbitrary units.

Supplementary Table 5. Evaluation of matrix effects in mouse brain tissue extracts and HEK293 cell lysates using a parallelism experiment.

Sample	% WT	Total α-synuclein assay				pS129 α-synuclein assay			
		Assay buffer		Equivalent dilution of KO matrix		Assay buffer		Equivalent dilution of KO matrix	
		Mean	SD	Mean	SD	Mean	SD	Mean	SD
10-fold diluted WT mouse brain tissue extract	0	116.0	2.9	733.0	75.0	110.0	11.6	111.0	7.6
	0.002	615.0	38.6	750.0	35.2	119.0	13.7	146.0	13.3
	0.005	2120.0	68.7	891.0	24.6	140.0	11.7	131.0	9.0
	0.01	6700.0	332.4	1878.0	18.5	158.0	9.0	140.0	19.7
	0.02	22715.0	1199.5	3747.0	332.0	167.0	13.0	140.0	8.1
	0.039	83573.0	1763.1	14633.0	848.6	204.0	23.2	142.0	9.5
	0.078	193758.0	4405.4	46588.0	1888.7	292.0	33.2	168.0	16.9
	0.156	328745.0	5403.2	108955.0	1519.8	432.0	36.4	242.0	23.2
	0.313	400612.0	19986.1	185878.0	17402.3	845.0	32.6	361.0	16.5
	0.625	430116.0	26996.0	276491.0	7605.2	2111.0	47.7	836.0	66.7
	1.25	462654.0	6127.1	315289.0	7690.5	4909.0	198.4	2120.0	92.2
	2.5	480020.0	16069.9	336507.0	11076.3	12329.0	341.7	5041.0	126.6
	5	476283.0	19863.4	324506.0	36664.0	22821.0	884.6	11551.0	449.6
	10	477771.0	9722.3	338408.0	10046.1	39129.0	2027.7	23888.0	302.3
100-fold diluted WT mouse brain tissue extract	0	127	9.8	160	12.7	107	10.4	109	8.1
	0.0002	125	15.2	161	21.5	105	9.3	116	9.3
	0.0005	126	12.3	160	8.5	114	13.9	141	10.4
	0.001	144	25.1	216	35.4	96	7.1	117	12.3
	0.002	261	18.2	250	25.1	108	9	129	5.2
	0.0039	653	34.7	844	100.2	122	17.5	143	9.3
	0.0078	2620	163.4	2487	89.7	129	17.2	142	9.5
	0.0156	12577	226	11533	1315.6	148	7.5	148	9.5
	0.0313	51931	864.7	48368	3174.6	153	9.5	156	2.6
	0.0625	141494	1746.4	119837	1982.2	205	11.6	168	6.7
	0.125	259505	1965.6	238368	5006.3	285	19.3	274	14
	0.25	365752	6254.7	329902	10584.8	649	26.8	724	4.4
	0.5	404770	12546.8	409735	11360.5	1678	64.5	1837	40.5
	1	423557	6035.7	429215	9867.3	4655	194.8	4356	39.5
5-fold diluted WT HEK293 cell lysate	0	115	12.1	261	30.5	101	13.3	83	7.1
	0.02	115	4.5	259	35.6	107	18.5	85	2.3
	0.039	134	0	300	32.4	118	16.2	83	7.1
	0.078	143	7.8	305	32.4	118	16.5	82	0.6
	0.156	154	18.2	360	65.2	176	22.7	113	22.5
	0.313	215	9.8	453	9.8	379	66.3	233	25
	0.625	493	39.9	794	73.8	1279	73.8	841	67.4
	1.25	1972	44.2	2496	81.3	5359	167.1	3471	437.6
	2.5	9483	101.7	8827	962.3	16345	1238.8	14320	497
	5	37689	890.2	34688	1512.8	49653	928.2	44995	1539.9
	10	117415	5111.7	98065	7460.8	128337	2064.5	108777	2346.6
	20	236526	5666.1	194142	3775.1	240675	3468.9	202285	2881.7
20-fold diluted WT HEK293 cell lysate	0	120	9	123	21	90	13.3	87	16.5
	0.005	108	9.7	120	18.5	85	2.3	88	11.6
	0.01	112	30.8	124	20.3	85	7.1	80	5.2
	0.02	109	17.4	113	20.6	91	8.1	75	6.7
	0.039	121	7.2	139	34.6	94	10.4	82	12.1
	0.078	110	15.6	139	23.6	87	11	92	11.5
	0.156	125	9.3	143	25.6	110	5.2	83	16.1
	0.313	150	19.7	168	2.9	148	18.7	118	9.5
	0.625	222	34	252	11.7	343	9.8	270	33.6
	1.25	467	33.6	555	29	1182	295.8	1140	104.2
	2.5	1856	68	2075	99.8	4855	249.5	5233	280.3
	5	10754	180.9	9879	347.9	15518	420.5	15014	152.7

Matrix effects produced by mouse brain matrix and HEK293 cell lysate were measured for new total (#ALSU-TASYN-B, Revvity) and pS129 (#ALSU-PASYN-B, Revvity) α-synuclein

assays using wild-type (WT) and α-synuclein knock-out (KO) mouse brain tissue extracts that had both been diluted 10-fold and 100-fold, as well as WT and α-synuclein KO HEK293 cell lysates that had both been diluted 5-fold and 20-fold. Data represent raw assay signal measurements in arbitrary units. SD, standard deviation.

Supplementary Table 6. Evaluation of mouse brain tissue matrix effects in total α-synuclein assay using a spike-recovery experiment.

Mouse WT α-synuclein spike concentration (pg/mL)	Wild-type brain		Knock-out brain		Assay buffer	
	Measured α-synuclein concentration	% recovery	Measured α-synuclein concentration	% recovery	Measured α-synuclein concentration	% recovery
10	3.07	30.65	8.25	82.51	8.91	89.12
20	9.58	47.88	17.84	89.21	16.40	81.99
40	39.26	98.15	39.56	98.89	38.07	95.16
80	70.41	88.01	74.15	92.68	76.78	95.98
160	138.47	86.54	157.53	98.46	164.26	102.66
320	296.74	92.73	316.50	98.91	316.03	98.76
640	775.55	121.18	668.75	104.49	894.59	139.78

Wild-type mouse α-synuclein was spiked into WT and *Snca* KO mouse brain tissue extracts diluted 2000-fold and 100-fold, respectively, as well as assay buffer (AB), to final concentrations ranging between 10-640pg/mL. Spike recovery was assessed using the new total α-synuclein assay (#ALSU-TASYN-B, Revvity) and a standard curve generated for purified mouse WT α-synuclein. Assay signal data are expressed as arbitrary units.

Supplementary Table 7. Evaluation of mouse brain tissue matrix effects in pS129 α-synuclein assay using a spike-recovery experiment.

Mouse pS129 α-synuclein spike concentration (pg/mL)	Wild-type brain		Knock-out brain		Assay buffer	
	Measured α-synuclein concentration	% recovery	Measured α-synuclein concentration	% recovery	Measured α-synuclein concentration	% recovery
0.625	0.33	52.40	0.53	84.65	0.50	79.56
1.25	1.04	83.50	1.28	102.47	1.31	104.49
2.5	1.82	72.99	2.14	85.53	2.30	92.18
5	4.04	80.80	4.16	83.12	4.39	87.87
10	9.10	91.05	9.63	96.27	9.07	90.71
20	16.77	83.84	17.72	88.58	19.24	96.21
40	44.67	111.68	42.62	106.55	44.25	110.63

Mouse pS129 α-synuclein was spiked into WT and *Snca* KO mouse brain tissue extracts diluted 2000-fold and 100-fold, respectively, as well as assay buffer (AB), to final concentrations ranging between 0.625-40pg/mL. Spike recovery was assessed using the new pS129 α-synuclein assay (#ALSU-PASYN-B, Revvity) and a standard curve generated for purified mouse pS129 α-synuclein. Assay signal data are expressed as arbitrary units.

Supplementary Table 8. Complete statistical test details.

Variable	Figure/panel	Data transformation	Two way ANOVA (Variable (F, DF, *p*))	Sidak's pairwise comparisons (*p* value)
ng pS129 α-synuclein/mg total protein	4c	Y = log(Y)	Brain region (230.7, 9, <0.0001)<br>Treatment (161.7, 1, <0.0001)	Sham vs PFF: MC (<0.0001), ACC (<0.0001), SSC (<0.0001), AMG (<0.0001), OLF (>0.9999), STR (0.3221), HIP (<0.0001), MB (<0.0001), MRN (0.9989), CB (0.9766)
ng total α-synuclein/mg total protein	4d	Y = log(Y)	Brain region (150.5, 9, <0.0001)<br>Treatment (180.1, 1, <0.0001)	Sham vs PFF: MC (<0.0001), ACC (<0.0001), SSC (<0.0001), AMG (<0.0001), OLF (0.0107), STR (<0.0001), HIP (0.2638), MB (0.0526), MRN (0.0001), CB (0.0013)
% α-synuclein phosphorylated at S129	4e	Y = log(Y)	Brain region (31.65, 9, <0.0001)<br>Treatment (617.5, 1, <0.0001)	Sham vs PFF: MC (<0.0001), ACC (<0.0001), SSC (<0.0001), AMG (<0.0001), OLF (0.0613), STR (<0.0001), HIP (<0.0001), MB (<0.0001), MRN (0.1155), CB (0.1166)
% total α-synuclein in PBS fraction	5a	None	Pathological burden (76.97, 2, <0.0001)<br>Treatment (5.334, 1, 0.0222)	Sham vs PFF: High (<0.0001), Moderate (0.9949), No (>0.9999)
% total α-synuclein in TrX fraction	5b	None	Pathological burden (68.93, 2, <0.0001)<br>Treatment (3.704, 1, 0.0560)	Sham vs PFF: High (0.0007), Moderate (>0.9999), No (>0.9999)
% total α-synuclein in SDS fraction	5c	None	Pathological burden (39.14, 2, <0.0001)<br>Treatment (60.32, 1, <0.0001)	Sham vs PFF: High (<0.0001), Moderate (<0.0001), No (>0.9999)
% pS129 α-synuclein in PBS fraction	5d	None	Pathological burden (36.00, 2, <0.0001)<br>Treatment (114.5, 1, <0.0001)	Sham vs PFF: High (<0.0001), Moderate (<0.0001), No (0.9998)

% pS129 α-synuclein in TrX fraction	5e	None	Pathological burden (94.36, 2, <0.0001)<br>Treatment (100.8, 1, <0.0001)	Sham vs PFF: High (<0.0001), Moderate (0.4267), No (0.9565)
% pS129 α-synuclein in SDS fraction	5f	None	Pathological burden (24.75, 2, <0.0001)<br>Treatment (93.31, 1, <0.0001)	Sham vs PFF: High (<0.0001), Moderate (<0.0001), No (>0.9999)
Monomeric pS129 α-synuclein (AU)	6a	None	Pathological burden (193.1, 2, <0.0001)<br>Treatment (13.18, 1, 0.0007)	Sham vs PFF: High (<0.0001), Moderate (0.5158), No (0.9167)
Multimeric pS129 α-synuclein (AU)	6b	None	Pathological burden (88.81, 2, <0.0001)<br>Treatment (52.58, 1, <0.0001)	Sham vs PFF: High (<0.0001), Moderate (0.7759), No (>0.9999)
Total α-synuclein (AU)	6c	None	Pathological burden (288.9, 2, <0.0001)<br>Treatment (9.949, 1, 0.0029)	Sham vs PFF: High (0.0874), Moderate (0.0003), No (0.6697)
Aggregated α-synuclein (ng/mg total protein) - PBS-soluble	6e	Y = log(Y)	Brain region (20.86, 9, <0.0001)<br>Treatment (105.3, 1, <0.0001)	Sham vs PFF: MC (<0.0001), ACC (<0.0001), SSC (<0.0001), AMG (0.0002), OLF (>0.9999), STR (>0.9999), HIP (>0.9999), MB (0.9829), MRN (>0.9999), CB (0.9370)
Aggregated α-synuclein (ng/mg total protein) - TrX-soluble	6f	Y = log(Y)	Brain region (8.844, 9, <0.0001)<br>Treatment (81.21, 1, <0.0001)	Sham vs PFF: MC (<0.0001), ACC (<0.0001), SSC (<0.0001), AMG (0.0013), OLF (0.9900), STR (0.6832), HIP (0.9995), MB (0.9017), MRN (0.9883), CB (>0.9999)

ACC, anterior cingulate cortex; AMG, amygdala; AU, arbitrary units; CB, cerebellum; DMB, dorso-medial midbrain; HIP, hippocampus; MC, motor cortex; OLF, olfactory bulb; PBS, phosphate-buffered saline; PFF, pre-formed fibril; SDS, sodium dodecyl sulfate; SSC, somatosensory cortex; STR, striatum; TrX, triton X-100; VMB, ventral midbrain.

Supplementary Table 9. Percentage of total α-synuclein recovered in PBS, Triton and SDS brain tissue fractions from PFF and sham mice.

Brain region	Pathological burden	Treatment	PBS			TrX			SDS		
			Mean	SD	n	Mean	SD	n	Mean	SD	n
MC	Severe	PFF	93.85	1.47	9	5.67	1.47	9	0.49	0.17	9
MC	Severe	Sham	96.62	0.92	8	3.33	0.92	8	0.05	0.06	8
ACC	Severe	PFF	88.41	4.34	9	10.76	3.99	9	0.83	0.47	9
ACC	Severe	Sham	92.42	1.75	8	7.51	1.79	8	0.07	0.08	8
SSC	Severe	PFF	90.51	2.74	9	8.71	2.56	9	0.78	0.31	9
SSC	Severe	Sham	92.90	2.21	8	6.42	1.59	8	0.18	0.03	8
AMG	Severe	PFF	91.77	1.82	9	7.76	1.76	9	0.47	0.15	9
AMG	Severe	Sham	94.52	0.42	8	5.40	0.47	8	0.08	0.08	8
OLF	Moderate	PFF	91.81	1.38	9	8.19	1.38	9	0.00	0.00	9
OLF	Moderate	Sham	93.24	1.65	8	6.76	1.65	8	0.00	0.00	8
STR	Moderate	PFF	94.72	1.12	9	5.05	1.12	9	0.23	0.03	9
STR	Moderate	Sham	93.27	1.27	8	6.55	1.24	8	0.18	0.04	8
HIP	Moderate	PFF	99.24	0.10	8	0.33	0.13	9	0.46	0.06	8
HIP	Moderate	Sham	99.39	0.14	8	0.21	0.10	8	0.40	0.07	8
VMB	Moderate	PFF	99.27	0.48	9	0.29	0.14	9	0.44	0.36	9
VMB	Moderate	Sham	99.40	0.29	8	0.22	0.08	8	0.38	0.26	8
DMB	No	PFF	99.83	0.05	8	0.17	0.05	8	0.00	0.00	9
DMB	No	Sham	99.90	0.02	8	0.10	0.02	8	0.00	0.00	8
CB	No	PFF	99.79	0.11	8	0.21	0.11	8	0.00	0.00	9
CB	No	Sham	99.83	0.11	8	0.17	0.11	8	0.00	0.00	8

ACC, anterior cingulate cortex; AMG, amygdala; CB, cerebellum; DMB, dorso-medial midbrain; HIP, hippocampus; MC, motor cortex; OLF, olfactory bulb; PFF, pre-formed fibril; SD, standard deviation; SSC, somatosensory cortex; STR, striatum; VMB, ventral midbrain.

Supplementary Table 10. Percentage of pS129 α-synuclein recovered in PBS, Triton and SDS brain tissue fractions from PFF and sham mice.

Brain region	Pathological burden	Treatment	PBS			TrX			SDS		
			Mean	SD	n	Mean	SD	n	Mean	SD	n
MC	Severe	PFF	74.77	6.89	9	6.06	2.20	9	19.17	7.14	9
MC	Severe	Sham	99.47	0.17	8	0.53	0.17	8	0.00	0.00	8
ACC	Severe	PFF	50.88	15.07	9	8.63	2.89	9	40.48	13.63	9
ACC	Severe	Sham	99.42	0.32	8	0.58	0.32	8	0.00	0.00	8
SSC	Severe	PFF	57.12	11.84	9	8.51	3.58	9	34.37	10.13	9
SSC	Severe	Sham	99.53	0.10	8	0.47	0.10	8	0.00	0.00	8
AMG	Severe	PFF	68.93	9.51	9	4.91	2.40	9	26.16	9.51	9
AMG	Severe	Sham	99.49	0.14	8	0.51	0.14	8	0.00	0.00	8
OLF	Moderate	PFF	98.92	0.56	9	1.08	0.56	9	0.00	0.00	9
OLF	Moderate	Sham	99.73	0.12	8	0.27	0.12	8	0.00	0.00	8
STR	Moderate	PFF	79.56	8.58	9	1.16	0.48	9	19.29	8.62	9
STR	Moderate	Sham	99.06	0.53	8	0.94	0.53	8	0.00	0.00	8
HIP	Moderate	PFF	72.23	18.80	9	0.66	0.30	9	27.11	18.93	9
HIP	Moderate	Sham	99.58	0.19	8	0.42	0.19	8	0.00	0.00	8
VMB	Moderate	PFF	74.29	20.52	9	1.04	0.35	9	24.67	20.48	9
VMB	Moderate	Sham	99.69	0.16	8	0.31	0.16	8	0.00	0.00	8
DMB	No	PFF	99.38	0.19	8	0.57	0.22	9	0.00	0.00	8
DMB	No	Sham	99.74	0.13	8	0.26	0.13	8	0.00	0.00	8
CB	No	PFF	99.70	0.12	9	0.30	0.12	9	0.00	0.00	9
CB	No	Sham	99.86	0.10	8	0.14	0.10	8	0.00	0.00	8

ACC, anterior cingulate cortex; AMG, amygdala; CB, cerebellum; DMB, dorso-medial midbrain; HIP, hippocampus; MC, motor cortex; OLF, olfactory bulb; SD, standard deviation; SSC, somatosensory cortex; STR, striatum; VMB, ventral midbrain.

Supplementary Table 11. Concentration of total α-synuclein recovered in PBS, Triton and SDS brain tissue fractions from PFF and sham mice. Data represent ng total α-synuclein per mg total protein.

Brain region	Pathological burden	Treatment	PBS			TrX			SDS		
			Mean	SD	n	Mean	SD	n	Mean	SD	n
MC	Severe	PFF	259.33	50.49	9	94.53	26.22	9	7.05	2.51	9
MC	Severe	Sham	560.75	194.54	8	175.58	80.49	8	1.62	1.78	8
ACC	Severe	PFF	218.00	32.16	8	117.01	35.46	8	8.08	3.77	9
ACC	Severe	Sham	410.00	89.32	7	234.27	124.70	8	1.22	1.32	8
SSC	Severe	PFF	214.89	30.60	9	126.51	51.03	9	6.28	2.13	9
SSC	Severe	Sham	417.75	91.19	8	219.93	34.49	7	2.70	0.42	8
AMG	Severe	PFF	325.11	59.03	9	142.62	55.63	9	7.85	2.88	9
AMG	Severe	Sham	560.50	127.05	8	274.46	92.83	8	2.00	1.76	8
OLF	Moderate	PFF	253.11	49.27	9	143.15	65.78	9	0.00	0.00	9
OLF	Moderate	Sham	429.25	154.29	8	206.18	60.44	8	0.00	0.00	8
STR	Moderate	PFF	273.78	40.83	9	90.29	39.51	9	2.57	0.35	9
STR	Moderate	Sham	524.25	128.42	8	191.05	33.42	8	2.90	0.49	8
HIP	Moderate	PFF	177.78	24.67	9	2.92	1.28	9	2.43	0.80	9
HIP	Moderate	Sham	237.25	39.81	8	2.72	1.85	8	2.29	0.34	8
VMB	Moderate	PFF	138.22	29.45	9	1.86	0.72	9	1.96	1.75	9
VMB	Moderate	Sham	200.75	42.16	8	2.71	1.40	8	1.73	1.13	8
DMB	No	PFF	72.67	15.91	9	0.66	0.26	9	0.00	0.00	9
DMB	No	Sham	119.50	24.95	8	0.80	0.37	8	0.00	0.00	8
CB	No	PFF	48.00	12.17	9	0.37	0.16	8	0.00	0.00	9
CB	No	Sham	96.50	79.76	8	0.58	0.22	8	0.00	0.00	8

ACC, anterior cingulate cortex; AMG, amygdala; CB, cerebellum; DMB, dorso-medial midbrain; HIP, hippocampus; MC, motor cortex; OLF, olfactory bulb; SD, standard deviation; SSC, somatosensory cortex; STR, striatum; VMB, ventral midbrain.

Supplementary Table 12. Concentration of pS129 α-synuclein recovered in PBS, Triton and SDS brain tissue fractions from PFF and sham mice. Data represent ng pS129 α-synuclein per mg total protein.

Brain region	Pathological burden	Treatment	PBS			TrX			SDS		
			Mean	SD	n	Mean	SD	n	Mean	SD	n
MC	Severe	PFF	1.45	0.33	9	0.75	0.43	9	1.98	0.87	9
MC	Severe	Sham	0.91	0.28	8	0.05	0.03	8	0.00	0.00	8
ACC	Severe	PFF	1.10	0.14	8	1.25	0.70	9	4.67	3.12	9
ACC	Severe	Sham	0.74	0.21	8	0.03	0.01	8	0.00	0.00	8
SSC	Severe	PFF	0.97	0.17	9	0.87	0.45	9	2.24	1.18	9
SSC	Severe	Sham	0.64	0.22	8	0.02	0.01	8	0.00	0.00	8
AMG	Severe	PFF	1.34	0.33	9	0.45	0.17	9	2.43	1.21	9
AMG	Severe	Sham	0.90	0.29	8	0.04	0.02	8	0.00	0.00	8
OLF	Moderate	PFF	1.24	0.31	9	0.08	0.05	9	0.00	0.00	9
OLF	Moderate	Sham	1.50	0.66	8	0.03	0.01	8	0.00	0.00	8
STR	Moderate	PFF	0.73	0.11	9	0.07	0.04	9	0.71	0.29	9
STR	Moderate	Sham	0.74	0.21	8	0.04	0.02	8	0.00	0.00	8
HIP	Moderate	PFF	0.46	0.12	9	0.02	0.01	9	0.57	0.50	9
HIP	Moderate	Sham	0.31	0.07	8	0.01	0.00	8	0.00	0.00	8
VMB	Moderate	PFF	0.37	0.08	9	0.03	0.01	9	0.52	0.45	9
VMB	Moderate	Sham	0.25	0.08	8	0.01	0.00	8	0.00	0.00	8
DMB	No	PFF	0.13	0.02	9	0.00	0.00	9	0.07	0.22	9
DMB	No	Sham	0.13	0.02	8	0.00	0.00	8	0.00	0.00	8
CB	No	PFF	0.08	0.01	9	0.00	0.00	9	0.00	0.00	9
CB	No	Sham	0.10	0.07	8	0.00	0.00	8	0.00	0.00	8

ACC, anterior cingulate cortex; AMG, amygdala; CB, cerebellum; DMB, dorso-medial midbrain; HIP, hippocampus; MC, motor cortex; OLF, olfactory bulb; SD, standard deviation; SSC, somatosensory cortex; STR, striatum; VMB, ventral midbrain.

Supplementary Table 13. Amount of α-synuclein aggregation quantified in PBS and Triton brain tissue fractions from PFF and sham mice using the LEGEND MAX α-Synuclein Aggregate ELISA.

Brain region	Pathological burden	Treatment	PBS			TrX		
			Mean	SD	n	Mean	SD	n
MC	Severe	PFF	15.96	8.82	9	19.06	10.61	9
MC	Severe	Sham	N.D.	N.D.	0	N.D.	N.D.	0
ACC	Severe	PFF	12.49	5.45	9	22.27	14.22	9
ACC	Severe	Sham	0.68	N.D.	1	N.D.	N.D.	0
SSC	Severe	PFF	14.44	4.16	9	30.14	16.06	9
SSC	Severe	Sham	0.32	N.D.	1	N.D.	N.D.	0
AMG	Severe	PFF	5.24	5.05	7	8.40	7.20	8
AMG	Severe	Sham	0.17	N.D.	1	2.25	N.D.	1
OLF	Moderate	PFF	11.27	N.D.	1	1.82	1.17	4
OLF	Moderate	Sham	N.D.	N.D.	0	1.09	N.D.	1
STR	Moderate	PFF	0.81	N.D.	1	2.88	3.08	7
STR	Moderate	Sham	N.D.	N.D.	0	0.28	N.D.	1
HIP	Moderate	PFF	N.D.	N.D.	0	2.71	3.77	3
HIP	Moderate	Sham	N.D.	N.D.	0	2.89	N.D.	1
VMB	Moderate	PFF	9.52	13.43	2	1.41	1.90	7
VMB	Moderate	Sham	0.05	N.D.	1	1.21	N.D.	1
DMB	No	PFF	1.23	N.D.	1	N.D.	N.D.	0
DMB	No	Sham	N.D.	N.D.	0	N.D.	N.D.	0
CB	No	PFF	N.D.	N.D.	0	0.90	N.D.	1
CB	No	Sham	N.D.	N.D.	0	0.57	N.D.	1

Mean and standard deviation (SD) values represent ng α-synuclein/mg total protein, while *n* represents the number of samples registering signals above the assay's lower limit of quantification (0.015ng/mL). Some regions had no samples with detectable aggregated α-synuclein, which were subsequently marked as not determined (N.D.). Standard deviation could not be determined in regions with less than 2 measurements. ACC, anterior cingulate cortex; AMG, amygdala; CB, cerebellum; DMB, dorso-medial midbrain; HIP, hippocampus; MC, motor cortex; OLF, olfactory bulb; SSC, somatosensory cortex; STR, striatum; VMB, ventral midbrain.

Supplementary Table 14. Sham and PFF mouse brain tissue extract dilutions prior to SureFire Ultra assay measurement.

Assay	Mouse model	Treatment	Tissue fraction	Fold dilution
**Total**	Wild-type	Sham	Homogenate	1000
	Wild-type	Sham	PBS	1000
	Wild-type	Sham	TrX	500
	Wild-type	Sham	SDS	50
	Wild-type	PFF	Homogenate	1000
	Wild-type	PFF	PBS	1000
	Wild-type	PFF	TrX	500
	Wild-type	PFF	SDS	50
**pS129**	Wild-type	Sham	Homogenate	100
	Wild-type	Sham	PBS	100
	Wild-type	Sham	TrX	50
	Wild-type	Sham	SDS	50
	Wild-type	PFF	Homogenate	100
	Wild-type	PFF	PBS	100
	Wild-type	PFF	TrX	50
	Wild-type	PFF	SDS	50

Whole tissue homogenates, as well as PBS-soluble, TrX-soluble and SDS-soluble tissue fractions, from sham and PFF mice were pre-diluted prior to measurement of total and pS129 α-synuclein using new SureFire Ultra assays to ensure values were within the linear dynamic range of each assay.
